# *Sanguisorba officinalis* L. ethanolic extracts and essential oil – chemical composition, antioxidant potential, antibacterial activity, and *ex vivo* skin permeation study

**DOI:** 10.3389/fphar.2024.1390551

**Published:** 2024-09-02

**Authors:** Anna Muzykiewicz-Szymańska, Anna Nowak, Edyta Kucharska, Krystyna Cybulska, Adam Klimowicz, Łukasz Kucharski

**Affiliations:** ^1^ Department of Cosmetic and Pharmaceutical Chemistry, Faculty of Health Sciences, Pomeranian Medical University in Szczecin, Szczecin, Poland; ^2^ Department of Chemical Organic Technology and Polymeric Materials, Faculty of Chemical Technology and Engineering, West Pomeranian University of Technology, Szczecin, Poland; ^3^ Department of Bioengineering, Faculty of Environmental Management and Agriculture, West Pomeranian University of Technology, Szczecin, Poland

**Keywords:** antioxidant activity, great burnet, HPLC, phenolic acid, GC-MS, Franz diffusion cell

## Abstract

**Introduction:**

*Sanguisorba officinalis* L. is classified as a medicinal plant and used in traditional medicine. The root of this plant is mainly used as a medicinal raw material, but the above-ground parts are also a valuable source of health-promoting biologically active compounds.

**Method:**

The study aimed to evaluate the antioxidant activity and total polyphenol content (TPC) of extracts prepared in 70% and 40% aqueous ethanol solution (dry extract content 50–500 g/L) from the aerial parts of *S. officinalis*. The essential oil was isolated from the tested raw material, and its composition was determined using GC-MS. Ethanolic extracts and essential oil have been tested for antibacterial activity. The extract in 70% *v/v* ethanol (dry extract content: 500 g/L) was subjected to HPLC analysis for the content of selected phenolic acids and an *ex vivo* skin permeation study. The ability of these metabolites to permeate and accumulate in the skin was analysed.

**Results:**

Extracts prepared at both ethanol concentrations showed similar antioxidant activity and TPC. Depending on the method, concentration of solvent, and dry extract content (50–500 g/L), the activity ranged from 1.97 to 84.54 g Trolox/L. TPC range of 3.80–37.04 g GA/L. Gallic acid (424 mg/L) and vanillic acid (270 mg/L) had the highest concentrations among the phenolic acids analysed. Vanillic acid (10 μg) permeates the skin at the highest concentration. The highest accumulation in the skin was found for 2,5-dihydroxybenzoic acid (53 μg/g skin), 2,3-dihydroxybenzoic acid (45 μg/g skin), and gallic acid (45 μg/g skin). The tested ethanolic extracts exhibited antibacterial activity. Samples with a dry extract concentration of 500 g/L showed the largest growth inhibition zones. The most sensitive strains to these extracts were *P. aeruginosa* (24 mm), *S. lutea* (23 mm), and *S. pneumoniae* (22 mm). The smallest inhibition zones were observed for *B. subtilis* (17 mm). The essential oil showed weaker antimicrobial activity (growth inhibition zone 8–10 mm). The GC-MS method identified 22 major components of the essential oil, including aliphatic hydrocarbons, unsaturated terpene alcohols, aliphatic aldehydes, unsaturated and saturated fatty acids, sesquiterpene, phytyl ester of linoleic acid, nitrogen compound, phytosterol, terpene ketone, phenylpropanoids, aliphatic alcohol, diterpenoid, aromatic aldehyde, and aliphatic carboxylic acid.

**Discussion:**

The conducted research has shown that ethanolic extracts from *Sanguisorbae herba* are a valuable source of compounds with antibacterial and antioxidant potential, including phenolic acids. The fact that selected phenolic acids contained in the tested extract have the ability to permeate and accumulate in the skin provides the basis for conducting extended research on the use of extracts from this plant raw material in cosmetic and pharmaceutical preparations applied to the skin.

## Graphical Abstract

**Figure:**
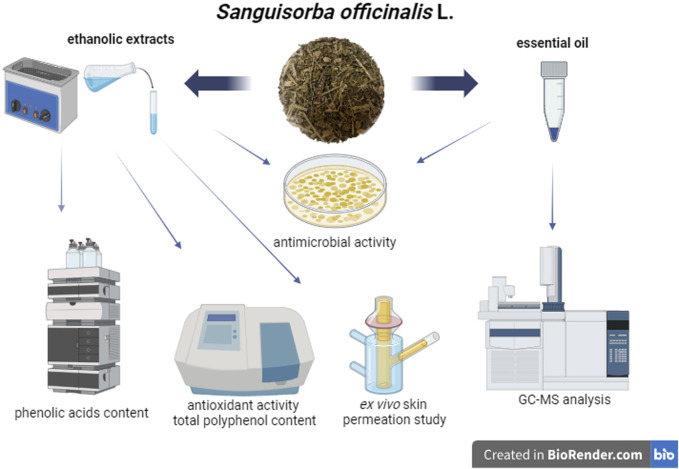

## 1 Introduction

Recently, there has been an increased interest in the use of plants and raw materials of plant origin as potential ingredients of drugs and oral supplements, as well as topically applied preparations, both pharmaceuticals and cosmetics. The reason for the increasing interest is, among others, the growing ecological awareness of consumers, as well as the frequent belief that plant preparations are healthier than those made from synthetic ingredients (Antignac et al., 2011; Fonseca-Santos et al., 2015; González-Minero and Bravo-Díaz, 2018; Nadeeshani Dilhara Gamage et al., 2022). One such interesting plant seems to be *S. officinalis* L., commonly called great burnet (Bunse et al., 2020). The genus *Sanguisorba* belongs to the Rosaceae family and includes 18 to 34 species and subspecies that are common in Europe, Asia, and North America. One of the most common species of the genus *Sanguisorba* is *S. officinalis* (Tocai et al., 2022). It is known as Zi-Yu in Japan and South Korea, Di-Yu in China, and Burnet in Western countries. This plant reaches a height of 30–120 cm and is characterized by pinnate leaves with a serrated margin, sturdy and spindle-shaped roots, as well as dark red flowers. The generative stage lasts from August to November. Traditional medicine uses this plant’s health-promoting properties. The roots (*Sanguisorbae radix*) and the aerial parts of the plant (*Sanguisorbae herba*) are used as botanical drugs. Traditional medicine uses the root of *S. officinalis* to heal wounds, cool blood, clear heat, and soothe snake bites. In Korea, great burnet root is used to regenerate the skin and alleviate inflammation, while the whole plant is used to treat hemorrhoids and female diseases. The aerial parts of this plant are also used to treat many diseases in Armenia. Many studies have confirmed the pharmacological activities of this vegetal product. The most frequently mentioned effects include anti-inflammatory, antimicrobial, antitumor, hemostatic and hematopoietic, hypoglycemic and lipid-lowering, neuroprotective, and antioxidant. *S. officinalis*, among others, uses its anti-inflammatory effect to treat airway inflammation (for example, bronchial asthma), contact and specific dermatitis, nephritis, and colitis (Zhou et al., 2021). Yu et al. (2011) and Yang et al. (2015) confirmed the anti-inflammatory effect of ethanol extracts from great burnet root, while Seo et al. (2016) and Yang et al. (2016) confirmed the anti-inflammatory effect of water extracts from *Sanguisorbae radix*. Su et al. (2018) suggest that phenolic compounds and linear monoterpenes are primarily responsible for the anti-inflammatory effect of *S. officinalis* root extracts (Su et al., 2018). In addition to its anti-inflammatory effect, great burnet has confirmed antimicrobial properties (Zhou et al., 2021). Tocai et al. (2022) confirmed the antibacterial effect of ethanolic crude extract from roots, leaves, and flowers against selected Gram-positive (*S. aureus*) and Gram-negative (*E. coli* and *P. aeruginosa*) strains (Tocai et al., 2022). Gawron-Gzella et al. (2016) showed the antibacterial effect of water and methanol extracts from the herb *S. officinalis*. The extracts showed activity against both Gram-positive (among others *S. aureus*, *S. epidermidis*, and *E. faecalis*) and Gram-negative (among others *E. coli* and *K. pneumoniae*) strains. In addition to their antibacterial activity, these extracts exhibited antifungal activity against *C. albicans* (Gawron-Gzella et al., 2016). Great burnet extracts also have proven antiviral activity. Kim et al. (2010) confirmed the anti-coronavirus effect of methanolic extracts from great burnet (Kim et al., 2010). Kim et al. (2022) also confirmed the antiviral effect of *S. officinalis* root extracts against enterovirus 71 (Kim et al., 2022). The anti-cancer potential of great burnet is also indicated (Zhou et al., 2021). Research has confirmed the antitumor potential of this plant in human gastric carcinoma (Zhu et al., 2013), human prostate cancer (Choi et al., 2012), and breast cancer (Wang et al., 2012). Great burnet (raw plant and charcoal) is known for its hemostatic effect. This plant also has hematopoietic properties. The extract of *S. officinalis* increases the number of white blood cells and reduces the bone marrow toxicity caused by antitumor treatments (Zhou et al., 2021). Son et al. (2015) indicate the potential of great burnet roots as a source of metabolites with antidiabetic effects due to, among others, lowering the level of glucose, insulin, and glycated hemoglobin in the blood. The authors also confirmed the protective effect of these metabolites on the kidneys and liver in patients with type 2 diabetes (Son et al., 2015). The neuroprotective effect of *Sanguisorbae radix* is described, among others, by Song et al. (2019). The authors describe the protective effect of Sanguiin H-11 on HT22 murine hippocampal cells against glutamate-induced death (Song et al., 2019). The therapeutic potential of *S. officinalis* in the treatment of other neurodegenerative disorders, such as Alzheimer’s disease or stroke, is also confirmed (Zhou et al., 2021). Many researchers (Byun et al., 2021; Lachowicz et al., 2020; Liao et al., 2008) confirmed the antioxidant potential of great burnet. The extracts (for example, aqueous, ethanolic, and methanolic) prepared from various parts of this plant (roots and aerial parts) showed this activity. The antioxidant potential of this plant may be the basis for many pharmacological activities (Zhou et al., 2021).

In addition to its medicinal effects, *S. officinalis* can be a valuable ingredient in cosmetic preparations. Both aerial parts and roots of great burnet are used in cosmetology. The *S. officinalis* root extract is classified as a cleansing, refreshing, skin conditioning, and tonic agent (Gawron-Gzella et al., 2016). Great burnet root extracts also indicate anti-wrinkle and moisturizing properties (Yoshida et al., 2019).

Species of the genus *Sanguisorba* contain numerous secondary metabolites with significant bioactive properties. Extracts (mainly methanolic and ethanolic) from *Sanguisorbae radix* and *Sanguisorbae herba* are a source of triterpenes, phenols, flavonoids, and other compounds (among others, neolignans, sterols, phenylpropanoid and monoterpenoid glycosides, saponins, lignans, and fatty acids) (Zhou et al., 2021). The high content of polyphenolic compounds (14,445 mg/100 g d.w. in flowers, 9,963 mg/100 g d.w. In leaves, 8,687 mg/100 g d.w. in roots, and 4,606 mg/100 g d.w. in stalks) suggests that this group of metabolites is responsible for the antioxidant activity of this plant. About 130 polyphenols were identified in the aerial parts and roots of *S. officinalis*. The following groups of polyphenolic compounds were identified in the leaves, flowers, stalks, and roots of great burnet: hydrolyzable tannins, sanguinis, sanguisorbic acids, phenolic acids, catechins, proanthocyanins, anthocyanins, and flavonols. The total content of hydrolyzable tannins in the leaves was 4,091 mg/100 g d.w., in the flowers 5,497 mg/100 g d.w., in the stalks 1,687 mg/100 g d.w., and in the roots it was 3,866 mg/100 g d.w. In all raw materials, the highest concentration was observed for Lambertianin C (3,029, 2,233, 899, and 1,237 mg/100 g d.w., respectively, for flowers, leaves, roots, and stalks). Sanguiin H-6 was found in the highest concentration in all raw materials (3,566, 621, 764, and 290 mg/100 g d.w., respectively, for flowers, leaves, roots, and stalks). The leaves had the highest concentration of sanguisorbic acids (116 mg/100 g d.w.), but the flowers did not contain them. In the leaves, the main compound belonging to this group was sanguisorbic acid glucoside (109 mg/100 g d.w.). Leaves were the richest source of phenolic acids (2,044 mg/100 g d.w.), primarily caffeoylquinic acid (1,364 mg/100 g d.w.). The content of this group of metabolites in flowers was 1,432 mg/100 g d.w., in stalks 361 mg/100 g d.w., while the roots contained less than 7 mg/100 g d.w. In stalks, similarly to leaves, the highest concentration was found for caffeoylquinic acid (183 mg/100 g d.w.), while in flowers, 5-caffeoylquinic acid (673 mg/100 g d.w.). Anthocyanins were identified only in flowers (550 mg/100 g d.w., mainly cyanidin 3-O-glucoside). All raw materials were sources of catechins and proanthocyanidins (1,889, 739, 3,239, and 1,208 mg/100 g d.w., respectively, in flowers, leaves, roots, and stalks). The main compound in this group of metabolites in all raw materials was B-type (epi)catechin dimmer. Flavonols were present primarily in leaves (1,970 mg/100 g d.w.). The concentration of these metabolites in flowers and stalks was at a similar level (718 and 788 mg/100 g d.w., respectively), while in leaves it was 48 mg/100 g d.w. In flowers, leaves, and stalks, the main flavonol was quercetin 3-O-glucuronide (Lachowicz et al., 2020).

Although *S. officilinalis* is a medicinal raw material with a long tradition and its composition is quite well researched, it is difficult to find in the available literature studies on penetration and accumulation in the skin its active compounds, including phenolic acids. There are also no studies about the composition of essential oils obtained from the vegetal product of great burnet. Therefore, the aim of the study was to evaluate the antioxidant activity (using DPPH^•^ and ABTS^•+^ methods) and total polyphenol content (using the Folin-Ciocalteu technique) of extracts prepared in 70% and 40% aqueous ethanol solution (dry extract content 50–500 g/L) prepared from *S. officinalis* herb (aerial part). The essential oil isolated through the distillation was analysed for composition using GC-MS. Extracts prepared in ethanol 70% and 40% *v/v* (dry extract content 50–500 g/L) and essential oil were analysed for antibacterial activity. The extract in 70% *v/v* ethanol (dry extract content: 500 g/L) was subjected to HPLC-UV analysis for the content of selected phenolic acids and an *ex vivo* skin permeation study. We assessed the ability of identified phenolic acids to permeate and accumulate in pig skin.

## 2 Material and methods

### 2.1 Reagents

2,2-diphenyl-1-picrylhydrazyl (DPPH^•^), 6-hydroxy-2,5,7,8-tetramethylchroman-2-carboxylic acid (Trolox), 2,2′-azino-bis (3-ethylbenzothiazoline-6-sulfonic acid) diammonium salt (ABTS), Folin–Ciocalteu reagent, gallic acid, were from Merck (Darmstadt, Germany). Sigma Aldrich (Darmstadt, Germany) provided the standards used for HPLC analysis. Ethanol, methanol, potassium persulfate, 99.5% acetic acid, sodium acetate anhydrous, and sodium carbonate were from Chempur (Piekary Śląskie, Poland). Aqueous ethanol solutions with a concentration of 70% and 40% *v/v* were used to prepare the extracts. All reagents were of analytical grade.

### 2.2 Plant material

The dried and cut aerial parts of *S. officinalis* (*Sanguisorbae herba,* size 0.5 to 1 cm) were purchased from the herbal store Aromatika Adam Iwańczuk (Hajnowka, Poland). According to the manufacturer’s declaration, the plant material was collected from conventional or natural crops in the Podlasie and Lublin regions (Poland). The portion of the plant material was deposited (No. SA-2022-01) in the place indicated in the affiliation of the corresponding author.

### 2.3 Extraction of plant raw material

The ethanolic extracts were prepared by ultrasound-assisted extraction (40 kHz, 45°C ± 1.0°C, 60 min) using the ultrasonic bath with a thermostat (FSF-031S, ChemLand, Poland). All extracts were filtered through Whatman filter paper No. 10. In the first stage of the research, to select the concentration of ethanol used to prepare extracts for further analyses, 5 g of the dried raw material was weighed in a conical flask, and 100 mL of ethanol at different concentrations was added as a solvent (Supplementary Table S1). In the second stage of the study, we prepared the extracts using 40 g of plant material and 160 mL of ethanol in water at a concentration of 40% or 70% *v/v* to assess the antioxidant and antimicrobial properties, conduct *ex vivo* skin permeation studies, and perform HPLC analysis. We used the same extract preparation technique as in the preliminary study. The obtained extracts were evaporated under reduced pressure at 57°C using a vacuum evaporator (INGOS s.r.o., Type RVO 400, Czech Republic). Before analyses, dry extracts were diluted with ethanol with the appropriate concentration (40% or 70% *v/v*) to obtain samples with a dry extract concentration of 50, 125, 250, and 500 g/L. The samples were stored in the dark at 4°C until further analysis.

### 2.4 Essential oil isolation

The essential oil was isolated in a steam distillation process using a Clevenger-type apparatus. The distillation was carried out until the last portion of the essential oil was obtained from the raw material (∼6 h). The oil was obtained in portions by distilling 50 g of the raw material in 500 mL of distilled water. Finally, all portions obtained in the 30 distillation series were mixed into one sample and dried over anhydrous sodium sulfate. The essential oil sample was stored in the dark at 4°C until analysis.

### 2.5 Antioxidant activity and total polyphenol content (TPC)

The antioxidant activity of extracts was assessed using the DPPH^•^ and ABTS^•+^ methods described by Nowak et al. (2023). The methodology described by these authors (Nowak et al., 2023) was also used to evaluate the total polyphenol content using the Folin-Ciocalteu technique. The results were calculated based on the curves determined for standard solutions of Trolox (DPPH^•^ and ABTS^•+^ methods) and gallic acid (Folin-Ciocalteu method). Curve equation for the DPPH^•^ method: y = −0.9795x + 0.9342 (*R* ^2^ = 0.977), for the ABTS^•+^ method: y = −0.2718x + 0.9324 (*R* ^2^ = 0.998), and for the Folin-Ciocalteu technique: y = −0.9947x – 0.0032 (*R* ^2^ = 0.998). In the DPPH^•^ and ABTS^•+^ methods, the results of antioxidant activity were expressed as g Trolox/L, while the total polyphenol content was presented as g gallic acid/L [g GA/L].

### 2.6 Antibacterial activity of extracts and essential oil

We analysed the antimicrobial properties of ethanolic extracts prepared using 40% and 70% *v/v* ethanol at dry extract concentrations of 50, 125, 250, and 500 g/L. The control sample was pure ethanol in both concentrations. The essential oil was used in concentrated form. Antibacterial properties were assessed for ten strains: *Sarcina lutea* ATCC 9341, *Enterococcus faecalis* ATCC 29212, *E. faecium*, *Escherichia coli*, *Pseudomonas fluorescens*, *P. aeruginosa*, *Streptococcus pneumoniae* ATCC 49619, *Bacillus coagulans*, *B. subtilis*, and *B. megaterium*. The sensitivity of test microorganisms to the tested extracts and essential oil was determined by the diffusion method into an agar medium using the well variant (Oke et al., 2013; Valgas et al., 2007). We used TSA (Tryptone Soya Agar) medium to cultivate the bacteria. The medium (20 mL) was poured into Petri dishes (⌀ 90 mm), and after solidification of the medium, 7 wells (⌀ 6 mm) were cut using a sterile cork borer. 0.1 mL of a 24-h bacterial culture in liquid tryptic soy medium (TSB) was placed on the Petri dishes. The inoculum was spread evenly over the surface of the medium using a glass smoother. The density of bacterial cultures ranged from 1 to 5∙10^7^ CFU/mL. The dishes with the sown strains were left to completely absorb the liquid for about 30 min, and then 10 µL of the tested extracts in various concentrations were introduced into individual wells. In the case of essential oil, sterile blank paper discs (⌀ 6 mm, BioMaxima, Poland) were placed on solidified substrates, and 10 μL of the tested substance was placed on them. Each experimental set was performed in three repetitions. Incubation of bacterial cultures was carried out for 72 h at 30°C, and *E. coli* bacteria were incubated at 37°C. The inhibitory effect of the tested samples was assessed based on the growth inhibition zone of the culture. Measurements were performed every 24 h, and the results after 72 h were used for final analyses.

### 2.7 GC-MS analysis of essential oil

Gas Chromatography-Mass Spectrometry (GC-MS) analysis was performed using a Shimadzu GC-MS-QP2020 NX (Shimadzu, San Jose, CA, United States) with a Shimadzu SH-I-5MS column (30 m × 0.25 mm × 0.25 μm). The column temperature was kept at 40°C for 2 min, and programmed to 300°C at a rate of 10°C/min and kept constant at 300°C for 2 min. Helium’s flow rate as a carrier gas is 35 cm/s (1 μL/min). MS were taken at 70 eV with split 10. The analysis duration was 30 min, and the sample volume was 1 µL. Identification of the constituents of the oil was made by comparison of their mass spectra located in the spectra library (NIST-2020). All samples were tested three times.

### 2.8 HPLC analysis

The concentration of tested metabolites in *S. officinalis* extract (dry extract content: 500 g/L prepared in 70% *v/v* ethanol) was determined by high-performance liquid chromatography (HPLC-UV), using the HPLC system from Knauer, Germany, according to the modified method described by Nowak et al. (2021a), Nowak et al. (2021b). The tested compounds were separated on a 125 mm × 4 mm column containing Eurospher, particle size 5 μm. The mobile phase consisted of a 1% aqueous solution of acetic acid and MeOH (120:10 by vol.), the flow rate was 1 mL/min 20 μL of the sample was injected into the column. The correlation coefficient of the calibration curve was 0.9999 for gallic acid (y = 1312425x – 3.4735, t_R_ – 5.450 min); 0.9997 for chlorogenic acid (y = 115454x + 3.9602, t_R_ – 44.704 min); 0.984 for 3,4-dihydroxybenzoic acid (y = 56067x + 27.274, t_R_ – 10.574 min); 0.9997 for 2,5-dihydroxybenzoic acid (y = 76485x – 1.4553, t_R_ – 14.567 min); 1.000 for 2,3-dihydroxybenzoic acid (y = 51612x + 1.4847, t_R_ – 21.167 min); 1.000 for caffeic acid (y = 210699x + 0.8081, t_R_ – 39.438 min); and 0.9998 for vanillic acid (y = 43645x + 3.1166, t_R_ – 19.027). All samples were analysed three times.

### 2.9 *Ex vivo* skin permeation study

The permeation experiments were performed using Franz diffusion cells (Phoenix DB-6, ABL&E-JASCO, Wien, Austria) according to the previously described methodology (Jacobi et al., 2007; Khiao et al., 2019). The Franz diffusion cell consists of a donor chamber with a capacity of 1 mL and an acceptor chamber with a capacity of 10 mL. The acceptor chambers were filled with a PBS solution (pH 7.4). During the experiment, a constant temperature of 37.0°C ± 0.5°C was maintained in each chamber. The buffer in the acceptor chamber was stirred (350 rpm). An extract prepared in 70% ethanol with a dry extract content of 500 g/L was used for the transdermal diffusion study. In the experiment, we used the abdominal part of porcine skin purchased from a local slaughterhouse. Skin samples with a thickness of 0.5 mm were cut with a dermatome, and then fragments with a diameter of 2 cm were cut out of them. The skin samples were wrapped in aluminum foil and stored at −20°C until use, but no longer than 3 months. This method of storage was safe for preserving the skin’s barrier properties (Badran et al., 2009). On the day of the experiment, the skin samples were slowly thawed at room temperature for 30 min and hydrated with PBS buffer pH 7.4 (Haq and Michniak-Kohn, 2018; Kuntsche et al., 2008; Simon et al., 2016). Undamaged pieces of skin (checked by measuring skin impedance) were placed between the donor and acceptor chambers. The impedance measurement verified its integrity. For this purpose, an LCR meter 4,080 (Voltcraft LCR 4080, Conrad Electronic, Germany), which was operated in parallel mode at an alternating frequency of 120 Hz (error at kΩ values < 0.5%), was used. The tip of the measuring probe was dipped in the donor and acceptor chambers and filled with PBS buffer (pH 7.4), as described previously by Kopečná et al. (2017) and Makuch et al. (2020). Only skin samples with impedance >3 kΩ were used. These values are similar to the electrical resistance of human skin (Davies et al., 2004). After placing the skin in the Franz diffusion cells, all chambers were allowed to equilibrate at 37°C for 15 min. Thereafter, a defined dose (1 mL) of the *S. officinalis* extract was applied to the skin’s outer side. All donor chambers were closed with plastic stoppers to prevent excessive evaporation of the solution. The experiment was conducted over 24 h. During this time, samples of the acceptor fluid (0.5 mL) were taken every hour (1–8 h of permeation and after 24 h from the beginning of the test), and the acceptor chamber was refilled with fresh PBS solution. The phenolic acid concentrations in the acceptor phase were measured by the HPLC method. The cumulative mass (µg) of each phenolic acid studied was calculated based on the obtained concentration. The antioxidant activity of the samples collected after completing the permeation study was also tested. After 24 h of experimentation, the diffusion cells were disassembled, and the skin samples were analysed for the content of selected phenolic acids. The accumulation of phenolic acids in the skin after permeation was determined using modification methods described by Haq and Michniak-Kohn (2018) and Janus et al. (2020). After 24 h of the experiment, each skin sample was removed and carefully rinsed in PBS (pH 7.4) (Kopečná et al., 2017). The skin was then cut around the diffusion area (1 cm^2^) and dried at room temperature. Each of the skin samples was cut into small pieces, placed in 2 mL of methanol, and incubated for 24 h at 4°C. After this time, skin samples were homogenized for 3 min using a homogenizer (IKA®T18 digital ULTRA TURRAX, Germany). The homogenate was centrifuged at 3,500 rpm (RCF 1776) for 5 min. The supernatant was analysed by HPLC. The accumulation of phenolic acids in the skin was calculated by dividing the amount of substances remaining in the skin by the mass of the skin sample and was expressed as the mass of phenolic acid per mass of the skin (μg/g).

### 2.10 Statistical analysis

The results are presented as the arithmetic mean ± standard deviation (SD) of the measurements of three independent samples. Pearson correlations (r) were determined between the results obtained using the DPPH^•^, ABTS^•+^, and Folin-Ciocalteu methods. The results of extracts prepared in 70% and 40% *v/v* ethanol were analysed using one-way analysis of variance (ANOVA, Tuckey test). The results of antimicrobial activity were assessed by a multivariate analysis of variance. Differences between the growth inhibition zones after using extracts with different dry extract contents (50–500 g/L) for individual strains were assessed using the Duncan test. Statistical analysis was performed using Statistica v.13.3 (StatSoft, Poland). The level of significance was α < 0.05.

## 3 Results

### 3.1 Antioxidant activity and total polyphenol content in extracts

**FIGURE 1:**
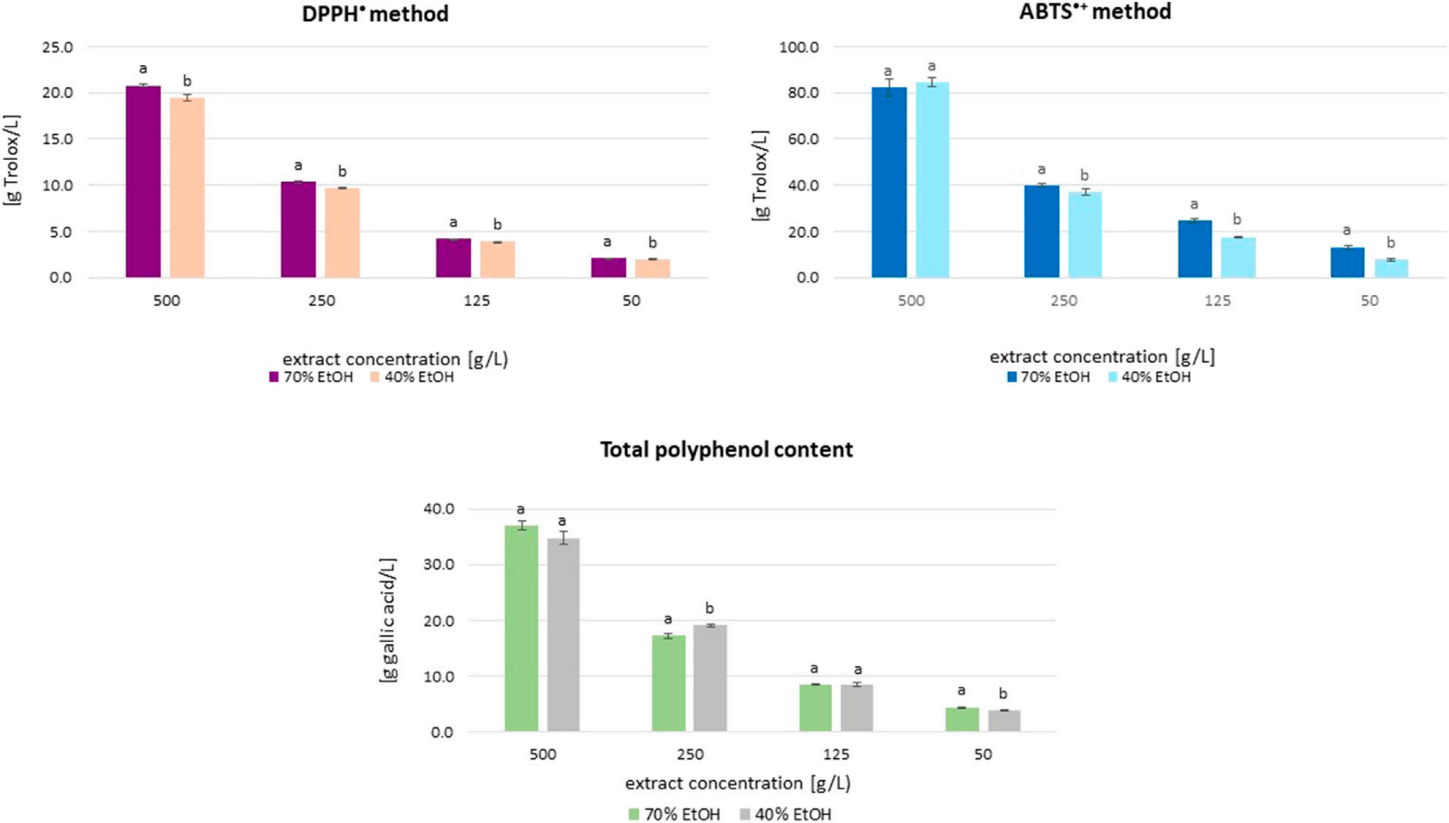
Mean (±SD) antioxidant activity and total polyphenol content (TPC) of *S. officinalis* ethanolic extracts in different concentration. Vertical lines represent standard deviation (SD), n = 3. Different letters mean significant differences between activity of extracts prepared in 70% and 40% ethanol.

The results of antioxidant activity (DPPH• and ABTS•+ methods) and total polyphenol content (TPC) in great burnet ethanolic extracts at various concentrations are presented in Figure 1. The antioxidant activity of the extracts assessed by the DPPH• method, depending on the extract concentration (50–500 g/L), was in the range of 2.08–20.76 g Trolox/L (extracts in 70% ethanol) and 1.97–19.46 g Trolox/L (extracts in 40% ethanol). In the case of activity assessed by the ABTS•+ method, these ranges were 13.11–82.39 g Trolox/L and 7.72–84.54 g Trolox/L, respectively. The total polyphenol content in extracts prepared with 70% ethanol was 4.37–37.04 g GA/L, whereas for samples prepared with 40% ethanol, it was 3.80–34.83 g GA/L. The extracts prepared using both ethanol concentrations showed similar antioxidant activity and total polyphenol content but generally differed statistically significantly (Figure 1). The exceptions were extracts at dry extract concentrations of 500 g/L (ABTS^•+^ method), as well as 125 and 500 g/L (TPC), for which the differences between the obtained results were statistically insignificant (*p* > 0.05). There were statistically significant correlations (r ≥ 0.991; *p* ≤ 0.0001) between the results from the different methods (DPPH•, ABTS•+, and F-C) for extracts in 70% and 40% ethanol (Supplementary Figures S1–S3). The results obtained using the ABTS•+ and DPPH• methods correlated statistically significantly with each other in the case of extracts prepared in 70% and 40% ethanol (r = 0.995 and r = 0.997; *p* ≤ 0.01, respectively). The concentration of polyphenolic compounds was significantly correlated with the antioxidant activity determined by the DPPH• and ABTS•+ methods (0.993–0.999; *p* < 0.01). We also observed statistically significant correlations between the activity of extracts prepared in 70% and 40% *v/v* ethanol, assessed using the same method (r = 0.99 (9), r = 0.999, and r = 0.995 (*p* < 0.01) for the DPPH•, ABTS•+, and F–C methods, respectively). By analysing the correlations between the results obtained by individual methods, regardless of the ethanol concentration used in the extraction process, correlation coefficients of r ≥ 0.991 and *p* < 0.0001 were obtained.

### 3.2 Antibacterial activity of extracts and essential oil

**FIGURE 2:**
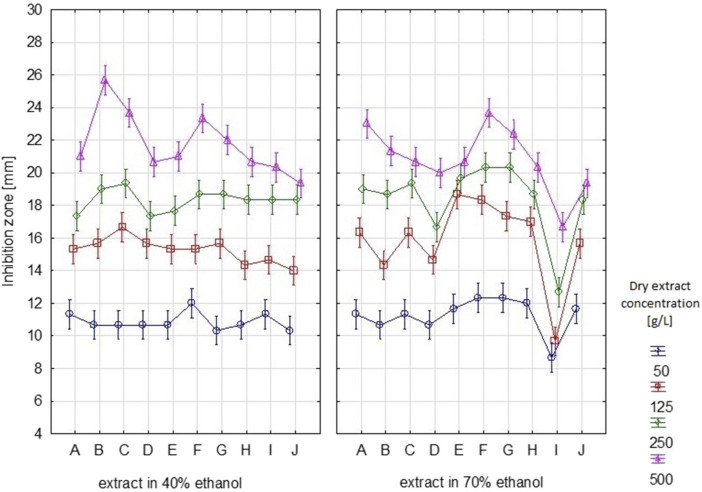
Growth inhibition zones [mm] for individual bacterial strains after using S*. officinalis* herb extracts prepared in 70% and 40% ethanol. (A – *S. lutea*, B – *E. faecalis,* C – *E. faecium*, D – *E. coli*, E – *P. fluorescens,* F – *P. aeruginosa,* G – *S. pneumoniae*, H – *B. coagulans,* I – *B. subtilis,* J – *B. megaterium).* Vertical lines represent standard deviation (SD).

**FIGURE 3:**
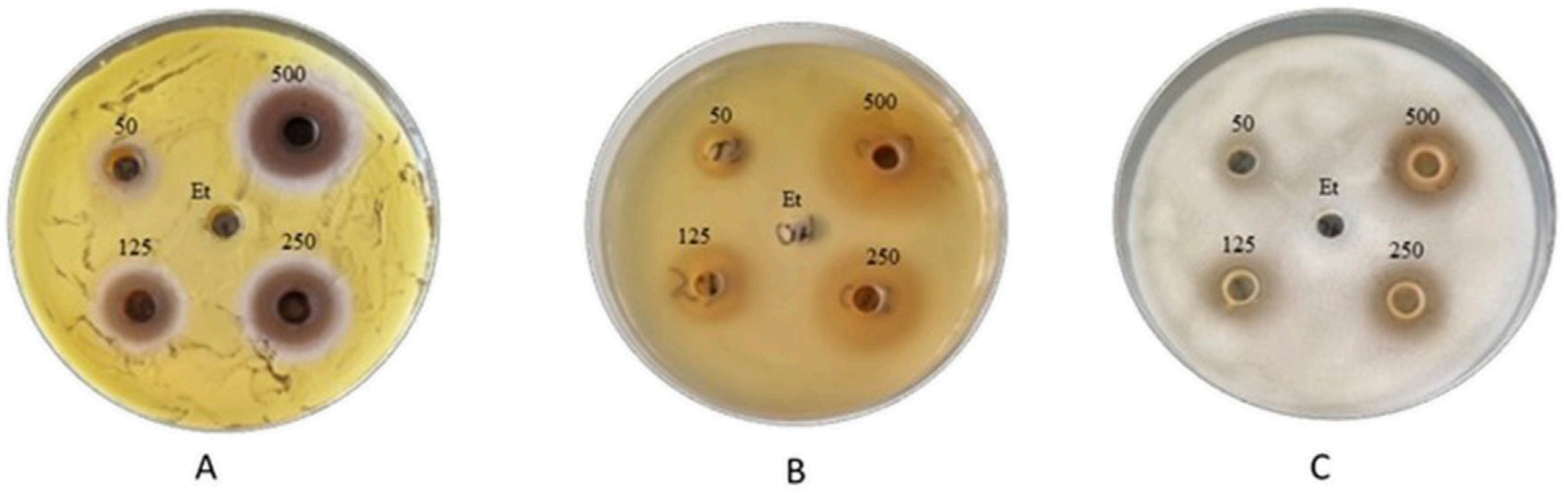
Zones of inhibition of the growth of **(A)** *S. lutea*, **(B)** *P. aeruginosa*, and **(C)** *B. subtilis* strains after using *S. officinalis* extract prepared in 70% ethanol.

The results of the antibacterial properties of the extracts are shown in Table 1 and Figure 2. Table 2 presents the results of the antibacterial activity of the essential oil. Figure 3 shows the growth inhibition zones of selected strains: *S. lutea*, *P. aeruginosa*, and *B. subtilis*.

**TABLE 1:** Antimicrobial activity of *S. officinalis* herb extract prepared in 70% ethanol.

Type of bacteria	Strain of bacteria	Dry extract concentration [g/L]			
		50	125	250	500
		Growth inhibition zone [mm]			
Gram-positive	*S. lutea*	11.3 ± 0.6^a^	16.3 ± 0.6^b^	19 ± 1^c^	23 ± 1^d^
	*E. faecalis*	10.7 ± 0.6^a^	14 ± 2^b^	18.7 ± 0.6^c^	21.3 ± 0.6^d^
	*E. faecium*	11.3 ± 0.6^a^	16 ± 2^b^	19.3 ± 0.6^c^	20.7 ± 0.6^c^
	*S. pneumoniae*	12.3 ± 0.6^a^	17.3 ± 0.6^b^	20.3 ± 0.6^c^	22.3 ± 0.6^d^
	*B. coagulans*	12 ± 1^a^	17 ± 1^b^	18.7 ± 0.6^c^	20.3 ± 0.6^d^
	*B. subtilis*	8.7 ± 0.6^a^	9.7 ± 0.6^a^	12.7 ± 0.6^b^	16.7 ± 0.6^c^
	*B. megaterium*	11.7 ± 0.6^a^	15.7 ± 0.6^b^	18.3 ± 0.6^c^	19 ± 1^c^
Gram-negative	*E. coli*	10.7 ± 0.6^a^	14.7 ± 0.6^b^	16.7 ± 0.6^c^	20 ± 1^d^
	*P. fluorescens*	11.7 ± 0.6^a^	18.7 ± 0.6^b^	19.7 ± 0.6^cd^	20.7 ± 0.6^d^
	*P. aeruginosa*	12.3 ± 0.6^a^	18.3 ± 0.6^b^	20.3 ± 0.6^c^	23.7 ± 0.6^d^

Values marked with the same letters (^a-d^) do not differ statistically significantly.

**TABLE 2:** Antibacterial activity of *S. officinalis* essential oil.

Strain of bacteria	Growth inhibition zone [mm]	Strain of bacteria	Growth inhibition zone [mm]
*B. megaterium*	10 ± 1	*S. lutea*	n.i
*P. aeruginosa*	9.5 ± 0.6	*S. pneumoniae*	n.i
*B. subtilis*	9.5 ± 0.6	*B. coagulans*	n.i
*E. coli*	8 ± 1	*E. faecalis*	n.i
*P. fluorescens*	n. i	*E. faecium*	n.i

n. i., no inhibition.

Regardless of the ethanol concentration used as a solvent, the analysed extracts inhibited or limited the growth of the tested bacterial strains, both Gram-positive and Gram-negative. The diameter of the growth inhibition zone depended on the concentration of the extract and the type of strain (Supplementary Table S2).

Depending on the dry extract concentration (50–500 g/L), regardless of the type of strain tested, growth inhibition ranged from 8.7 to 23.7 mm (Table 1). If we assume that inhibition zones less than 10 mm signify bacterial resistance to the tested substance, we can observe that *B. subtilis* exhibited the least sensitivity to the tested extract. For this strain, inhibition zones <10 mm were observed for samples with dry extract contents of 50 and 125 g/L. Depending on the content of the dry extract, the following strains were the most sensitive: *P. aeruginosa*, *S. pneumoniae*, *P. fluorescens,* and *S. lutea*.

After application of the essential oil, no zone of inhibition larger than 10 mm was observed for any tested strain.

### 3.3 Isolation and composition of *S. officinalis* essential oil

The distillation process had an efficiency of 0.04%. Table 3 displays the composition of the great burnet essential oil. We recognized 22 main components of the oil. The essential oil *of S. officinalis* consisted mainly of aliphatic hydrocarbons (2-Methylhexacosane, 1-Nonacosene, Triacontane, and Tetrapentacontane), unsaturated terpene alcohols (Linalool, Geraniol, and Phytol), aliphatic aldehydes (Eicosanal and Nonacosanal), unsaturated and saturated fatty acids (Dihomo-γ-linolenic and Undecanoic acids), sesquiterpene (Fragranyl acetate), phytyl ester of linoleic acid (Phytyl linoleate), nitrogen compound (N-hexatriacontane), phytosterol (Stigmastane-3,6-dione), terpene ketone (Farnesyl acetone), phenylpropanoids (Eugenol and Chavibetol), aliphatic alcohol (1-Nonen-3-ol), diterpenoid (Geranyl linalool), aromatic aldehyde (M-Tolualdehyde), and aliphatic carboxylic acid (10,12-Pentacosadiynoic acid).

**TABLE 3:** Composition of *S. officinalis* essential oil.

Class	Compound	Rt [min]	Ref.
aliphatic alcohol	1-Nonen-3-ol	8.191	Petri et al. (1991)
aromatic aldehyde	M-Tolualdehyde	9.740	Hamedeyazdan et al. (2013)
unsaturated terpene alcohol	Linalool	9.810	Esmaeili et al. (2010)
unsaturated terpene alcohol	Geraniol (2,6-Octadien-1-ol)	11.269	Silva et al. (2021), Guimarães et al. (2019)
sesquiterpene	Fragranyl acetate	11.690	Bader et al. (2022)
saturated fatty acid	Undecanoic acid (Lauric acid)	12.023	Bhuiyan et al. (2009)
phenylpropanoid	Eugenol	12.693	Srivastava et al. (2022)
phenylpropanoid	Chavibetol (3-Allyl-6-methoxyphenol)	14.051	Niculau et al. (2018)
terpene ketone	Farnesyl acetone	15.075	Sotubo et al. (2016)
unsaturated terpene alcohol	Phytol	15.250	Islam et al. (2018)
diterpenoid	Geranyl linalool	15.484	Sahoo et al. (2021)
aliphatic aldehyde	Eicosanal	15.959	Servi H (2019)
unsaturated fatty acid	Dihomo-γ-linolenic acid	18.573	Freije et al. (2013)
aliphatic hydrocarbons	2-Methylhexacosane	18.610	Ryu et al. (2020)
phytosterol	Stigmastane-3,6-dione	18.705	Radulovic and Djordjevic (2011)
aliphatic hydrocarbons	1-Nonacosene	18.829	Onocha et al. (2016)
aliphatic carboxylic acid	10,12-Pentacosadiynoic acid	21.990	Macias Socha and Reyes Cuellar (2020)
aliphatic aldehyde	Nonacosanal	22.075	Trivedi et al. (2022)
aliphatic hydrocarbons	Triacontane	26.645	Bouazzi et al. (2020)
phytyl ester of linoleic acid	Phytyl linoleate	27.103	Krauß et al. (2016)
nitrogen compound	N-hexatriacontane	28.117	Szymański et al. (2020)
aliphatic hydrocarbons	Tetrapentacontane	28.539	Değirmenci and Erkurt (2020)

### 3.4 HPLC analysis of *S. officinalis* extract

**FIGURE 4:**
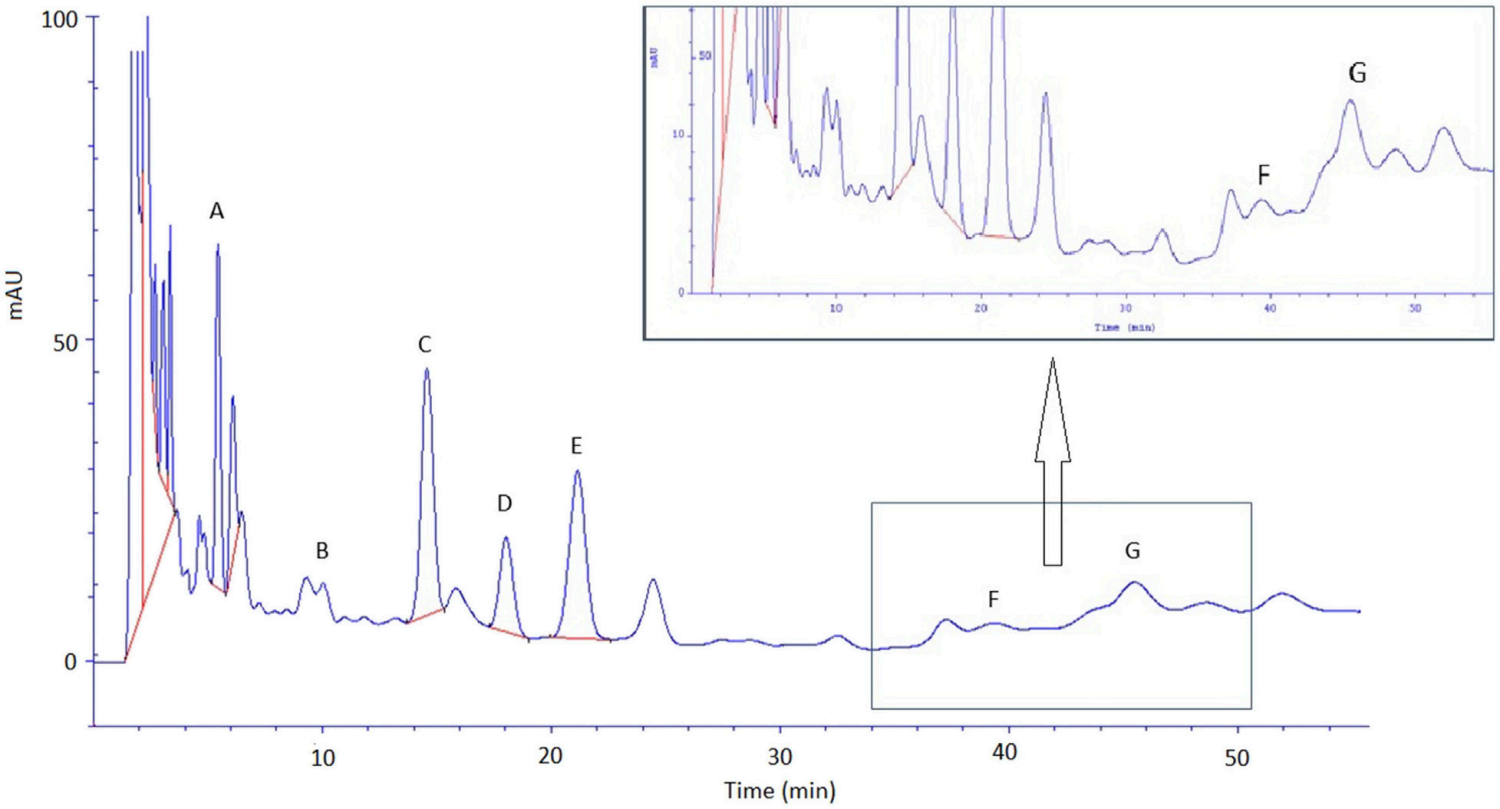
Chromatogram of phenolic acid identified in *S. officinalis* extract. A – gallic acid; B – 3,4-dihydroxybenzoic acid; C – 2,5-dihydroxybenzoic acid; D – vanillic acid; E – 2,3-dihydroxybenzoic acid, F – caffeic acid, and G – chlorogenic acid. Extracts were diluted 100-fold before HPLC analysis.

The great burnet extract prepared in 70% ethanol (dry extract concentration 500 g/L) was analysed by HPLC, and then it was used for an *ex vivo* skin permeation study. Table 4 presents the quantitative composition of the identified phenolic acids, while Figure 4 displays the chromatogram of this analysis. We identified the following phenolic acids in the great burnet extract: gallic acid, 3,4-dihydroxybenzoic acid, 2,5-dihydroxybenzoic acid, vanillic acid, 2,3-dihydroxybenzoic acid, caffeic acid, and chlorogenic acid. The highest concentration of the identified phenolic acids was found for gallic acid (424 ± 10 mg/L) and vanillic acid (270 ± 5 mg/L). The lowest concentration of the analysed phenolic acids was found for chlorogenic acid (13 ± 3 mg/L) and caffeic acid (21 ± 3 mg/L).

**TABLE 4:** The content (Mean ± SD) of phenolic acids in *S. officinalis* extract (n = 3).

Phenolic acid	Concentration [mg/L]
gallic acid	424 ± 10
3,4-dihydroxybenzoic acid	222 ± 9
2,5-dihydroxybenzoic acid	169 ± 15
vanillic acid	270 ± 5
2,3-dihydroxybenzoic acid	207 ± 7
caffeic acid	21 ± 3
chlorogenic acid	13 ± 3

### 3.5 *Ex vivo* skin permeation study

**FIGURE 5:**
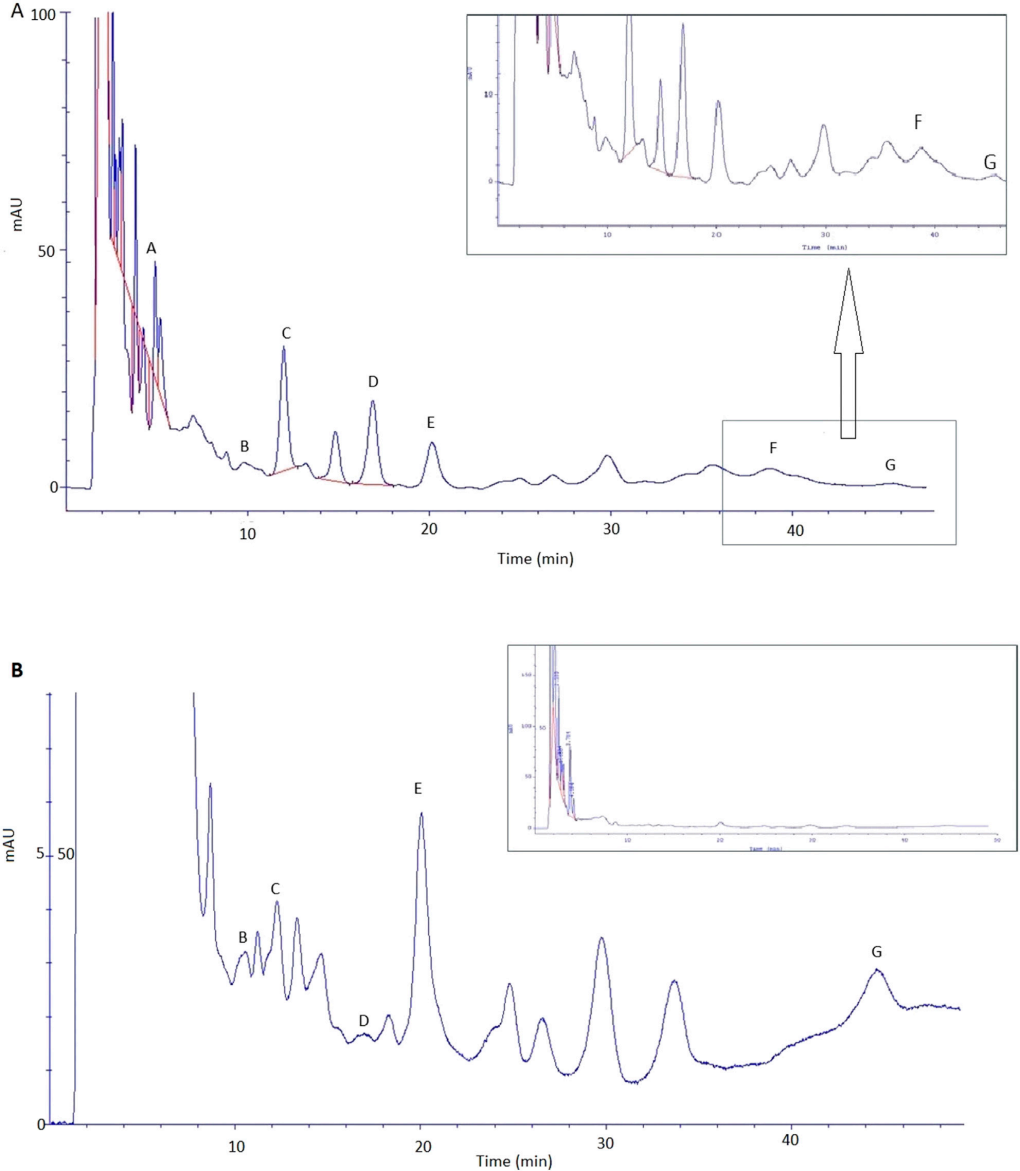
Chromatogram of phenolic acid identified in fluid after skin extraction **(A)** and acceptor fluid taken after 24 h of extract permeation **(B)**: A – gallic acid; B – 3,4-dihydroxybenzoic acid; C – 2,5-dihydroxybenzoic acid; D – vanillic acid; E – 2,3-dihydroxybenzoic acid, F – caffeic acid, and G – chlorogenic acid.

The extract prepared in 70% ethanol (dry extract concentration: 500 g/L) was used for transdermal diffusion studies. Table 5 shows the content of the analysed phenolic acids in the acceptor fluid after 24-h permeation and in the skin collected after the completion of the permeation of the great burnet extract. We did not identify any phenolic acids in the samples collected during the first 8 h of permeation. Therefore, we presented the results of samples collected after 24-h permeation. Figure 5 shows the HPLC chromatograms of the acceptor fluid after 24-h permeation (A) and the fluid obtained after skin extraction (B). Among the tested phenolic acids, vanillic acid permeated to a higher degree than others. Their cumulative content in the acceptor fluid after 24-h permeation was 10 ± 1 µg. The highest accumulation in the skin [µg/g skin] was observed for 2,5-dihydroxybenzoic acid (53 ± 4), 2,3-dihydroxybenzoic acid (45 ± 3), gallic acid (45 ± 3), and vanillic acid (31 ± 3) (Table 5).

**TABLE 5:** Mean (±SD) the content of phenolic acids in acceptor fluid and skin extract obtained after 24-h permeation study (n = 3).

Phenolic acid	Accumulation in the skin [µg/g skin]	Acceptor fluid after 24-h of permeation [µg]
gallic acid	45 ± 3	n.i
3,4-dihydroxybenzoic acid	21 ± 3	5.4 ± 0.7
2,5-dihydroxybenzoic acid	53 ± 4	1.6 ± 0.3
vanillic acid	31 ± 3	10 ± 1
2,3-dihydroxybenzoic acid	45 ± 3	3.7 ± 0.6
caffeic acid	3.2 ± 0.2	n.i
chlorogenic acid	2.0 ± 0.5	1.6 ± 0.3

n.i., not identified.

## 4 Discussion

The reports in the literature confirm the antioxidant activity of various parts of *S. officinalis*. Byun et al. (2021) assessed the antioxidant activity, total polyphenol, and flavonoid content in *S. officinalis* dry root extracts prepared using ethanol at different concentrations (20%, 40%, 80%, 100%). To prepare the extracts, they used a conventional reflux extraction technique. Extracts in 40% and 80% ethanol contained a similar concentration of polyphenols (121.96 mg GAE/g). The total flavonoid content was slightly higher in the extract prepared in 80% ethanol. Depending on the evaluation method, the antioxidant activity was generally the highest for extracts in 80% ethanol (TAC assay) and 40% alcohol (ABTS^•+^ and FRAP assay). In general, they obtained lower results (except for the DPPH^•^ method) for extracts prepared in 20% and 100% ethanol (Byun et al., 2021). The preliminary analysis of the presented research revealed a similar relationship. Extracts in 40% and 70% ethanol exhibited comparable high antioxidant activity and total polyphenol content, whereas extracts in 96% alcohol demonstrated significantly lower tested properties. Therefore, we conducted extended analyses on extracts prepared using 70% and 40% *v/v* ethanol. Many researchers emphasize the importance of ethanol concentration as a solvent in the extraction of biologically active compounds from plant material (Hikmawanti et al., 2021; Nour et al., 2013; Spigno et al., 2007). Their polarity is one of the factors influencing the differences in the tested potential of extracts prepared using different solvents (Freije et al., 2013; Herrera-Pool et al., 2021; Nawaz et al., 2020). Gawron-Gzella et al. (2016) compared the antioxidant activity (DPPH• method) and the total content of polyphenols, including flavonoids and phenolic acids, in aqueous and methanolic extracts from the powdered herb of *S. officinalis*. The IC_50_ determined by the DPPH• method for the methanolic extract was 2.95 mg/mL, while for the aqueous extract it was 3.65 mg/mL. Aqueous extracts were characterised by a higher total content of polyphenols and phenolic acids, while methanolic extracts contained more flavonoids (Gawron-Gzella et al., 2016). Nzor et al. (2024), based on studies of *Anthocleista vogelii* leaf extracts’ antioxidant activity, emphasize the importance of solvent polarity’s influence on the tested potential. These authors claim that polar solvents, such as ethanol or methanol, are more effective in extracting compounds with antioxidant potential than non-polar solvents, such as n-hexane or dichloromethane. For this reason, it seems important to optimise the extraction process, including the solvent used, for the specific group of biologically active compounds that we want to isolate from the tested plant material (Nzor et al., 2024). Lachowicz et al. (2020) compared the antioxidant activity of great burnet leaves, flowers, stalks, and roots. In their studies, leaf extracts were characterised by the highest antioxidant activity among all the tested morphological parts of the plant. The ABTS^•+^ method was used to measure the antioxidant activity of leaves, which was 6.63 mmol/g dry basis. This was statistically significantly different from the activity of flowers, roots, and stalks, which were 5.56, 5.08, and 0.52 mmol/g dry basis, respectively. The results obtained using the FRAP method showed a similar relationship. The iron ion reduction capacity for leaves, flowers, roots, and stalks, respectively, was 0.3, 0.2, 0.13, and 0.09 mmol/g on a dry basis (Lachowicz et al., 2020). Tocai et al. (2021) compared the antioxidant activity and TPC in extracts from different parts of two *Sanguisorba* varieties: *S. officinalis* (great burnet) and *S. minor* (small burnet). In their study, extracts from great burnet leaves were characterised by significantly higher antioxidant activity (assessed by DPPH^•^ and FRAP methods) and TPC than extracts from small burnet leaves. However, in this study, extracts from the roots of both varieties were the most valuable source of compounds with antioxidant potential, including polyphenols (Tocai et al., 2021). According to Pelc et al. (2011), these differences may be due to the lower content of tannins and phenolic acids in the herb *S. officinalis* compared to underground organs (Pelc et al., 2011). The results of our research and those of other authors indicate that both the aboveground and underground parts of *S. officinalis* may be a valuable source of compounds with antioxidant potential, including polyphenols. However, various factors, including the pre-extraction treatment of the raw material, solvent concentration, and extraction parameters, may significantly affect the activity of the extracts.

Tocai et al. (2022) assessed the antimicrobial activity of leaves, flowers, and root extracts in 70% ethanol prepared from *S. officinalis* and *S. minor*. They analysed three strains of bacteria: *S. aureus*, *E. coli*, and *P. aeruginosa*. *S. officinalis* leaf extracts (SOL) showed significantly higher activity than root (SOR) and flower (SOF) extracts. Depending on the strain, the diameter of the inhibition zone in the case of SOL was 8.4–11.5 mm, SOR was 5.5–6.6 mm, and SOF was 2.5–4.6 mm. In the case of great burnet extracts, the most sensitive strains were *P. aeruginosa* (inhibition zone 11.3 mm, 6.5 mm, and 4.6 mm, respectively for SOL, SOR, and SOF) and *E. coli* (11.5 mm, 6.6 mm, and 2.6 mm, respectively for SOL, SOR, and SOF). The antimicrobial activity of small burnet extracts was more varied, depending on the extracted raw material. The leaf extract had relatively strong antibacterial activity against *S. aureus* (inhibition zone 15.5 mm), while *E. coli* was most sensitive to root and flower extracts (inhibition zones 10.5 mm and 11.6 mm, respectively) (Tocai et al., 2022). In our study, the great burnet extracts also showed the strongest activity against *P. aeruginosa* (the inhibition zone, depending on the dry extract content in the sample, was 12.3–23.7 mm). The activity of the extracts against *E. coli* was also higher compared to the results obtained by Tocai et al. (2022) (the average inhibition zone was 15.5 mm) (Tocai et al., 2022). Do et al. (2005) analysed the antimicrobial activity of extracts prepared from selected medicinal plants. They evaluated the *S. officinalis* root extract prepared in 70% ethanol. Among the analysed Gram-positive strains, the extract showed activity against *B. subtilis* and *S. typhimurium* (MIC 62.5 and 61.5 μg/mL, respectively), while in the group of Gram-negative bacteria, it was mainly against *S. aureus*, *P. aeruginosa*, and *E. coli* (MIC for all strains was 62.5 μg/mL). However, the researchers noted no bactericidal properties against *L. plantarum* (Do et al., 2005). Gawron-Gzella et al. (2016) assessed the antimicrobial activity of aqueous and methanolic extracts from great burnet herb. These extracts showed similar antibacterial activity against Gram-positive strains (*S. aureus*, *S. epidermidis*, *M. luteus*, and *B. subtilis*—MIC for methanolic extract 0.7 mg/mL and aqueous extract 0.7–0.15 mg/mL) and Gram-negative strains (*E. coli*, *K. pneumoniae*—MIC for all extracts 0.3 mg/mL). In both extracts, they observed the lowest activity against *E. faecalis* (MIC 2.5 mg/mL). They used gentamicin as the reference antibiotic (the MIC, depending on the strain, ranged from 1 to 32 μg/mL) (Gawron-Gzella et al., 2016). In our study, *E. faecalis* was also one of the least sensitive strains. The average growth inhibition zone was 16.3 mm, which was the same as for *B. megaterium*. A smaller average zone of growth inhibition was observed only for *E. coli* (15.5 mm) and *B. subtilis* (12 mm), which was the least sensitive strain of all tested (Figure 2; Table 1). Zhu et al. (2019) evaluated the antibacterial activity of crude polyphenolic extract (CPE) and purified polyphenolic extract (PPE) prepared from *S. officinalis* in 70% ethanol. PPE showed higher antibacterial activity against both Gram-positive (*S. aureus*, *B. subtilis*, and *L. monocytogenes*) and Gram-negative bacteria (*E. coli* and *S. typhimurium*). The diameter of the growth inhibition zone of Gram-positive bacteria, depending on the strain, was 24.7–29.4 mm, while for Gram-negative strains it was 10.9–14.7 mm. These ranges for CPE were 13.9–15.2 mm and 7.2–10.6 mm, respectively. They observed the largest zones of growth inhibition for both PPE and CPE for *S. aureus* and the smallest for *E. coli* (Zhu et al., 2019). In our study, we also noted that *E. coli* was one of the least sensitive strains. The extraction method selection and the different content of plant material may cause differences in results. The content of biologically active ingredients in the raw material may also depend on the geographical zone of growth, the date of harvest, the method of processing the material after harvesting, and the plant’s pre-extraction treatment. The available literature contains no information regarding the antimicrobial activity of great burnet essential oil. However, it should be noted that the antimicrobial activity of essential oil is much lower than that of ethanolic extracts, and many of the tested strains are not sensitive to this essential oil. Some essential oils may have weak or no antimicrobial activity (Elhawary et al., 2021). The better and more effective antibacterial effect of alcohol extracts from *S. officinalis* is probably due to differences in the composition of active substances that limit the growth of the tested strains. The polyphenols in them may be responsible for the tested extracts’ antimicrobial potential. There are many reports in the literature confirming the strong antibacterial and antifungal properties of polyphenolic compounds (Daglia, 2012; Manso et al., 2021; Othman et al., 2019). Othman et al. (2019) emphasise that the antimicrobial potential of polyphenols is closely related to their antioxidant activity (Othman et al., 2019). In addition, some phenolic acids, e.g., gallic and caffeic acid, which were identified in the tested *S. officinalis* extracts, also have proven antibacterial properties. Their antimicrobial activity against strains of Gram-positive bacteria (e.g., *S. aureus* and *L. monocytogenes*) and Gram-negative bacteria (e.g., *E. coli* and *P. aeruginosa*) is indicated (Daglia, 2012).

A similar efficiency of the distillation process of essential oil from the herb *Epilobium angustifolium* L. (0.035%) was obtained by Nowak et al. (2021). In the research of Esmaeili et al. (2010) concerning the extraction of essential oil from *S. minor* they achieved a yield based on dry weight of 0.2% (w/w) (Esmaeili et al., 2010). No analysis of the composition of the essential oil of great burnet has been found in the literature, but analyses of the *S. minor* essential oil are available. In the *S. minor* essential oil, Wieteska et al. (2013) identified 33 components (60.52%). The relative amount (%) of the compounds were: nerol (13.87%), geraniol (5.70%), and phytol (5.16%). They also identified compounds that are classified as fragrances: lavandulol (3.07%), linalool (2.78%), hexahydrofarnesyl acetone (2.35%) (E)-β-Ionone (2.05%), eugenol (1.72%), farnesyl acetone (1.01%), and methyl cinnamate (0.40%) (Wieteska et al., 2013). Esmaeili et al. (2010) identified 17 compounds (93.2%) in the essential oil of *S. minor*, mainly aliphatic hydrocarbons (40.6%), five sesquiterpenes (36.8%), one oxygenated monoterpene (7.3%), and one aliphatic aldehyde (8.3%). The major components in this oil were (E, E) Farnesyl acetate (13.4%) – a terpene compound, nonadecane (11.2%), and docosane (11.0%), followed by β-caryophyllene (9.7%), nonanal (8.5%), and linalool (7.3%) (Esmaeili et al., 2010). Our *S. officinalis* essential oil contained similar classes of compounds (terpene ketone, aliphatic hydrocarbons, sesquiterpene, and aliphatic aldehyde) as the *S. minor* essential oil analysed by Esmaeili et al. (2010). Small burnet essential oil was richer in sesquiterpene, while great burnet essential oil contained more fatty acids (saturated and unsaturated). This comparison suggests that essential oils obtained from different varieties of the same plant may have variable chemical compositions, which may result in various biological activities. Unsaturated terpene alcohols and unsaturated fatty acids are the main components of the *S. officinalis* essential oil (Table 3) (Nowak et al., 2021a). The identified components of *S. officinalis* essential oil have a variety of biological effects. Linalool is a valuable molecule with significant therapeutic potential, making it a valuable marker in assessing the authenticity of aromas, fragrances, and essential oils. At concentrations of 0.1% (*v/v*), linalool exhibits antimicrobial activity against microorganisms such as *S. aureus*, *B. subtilis*, *E. coli*, and *P. multocida*, being more active against Gram-positive bacteria compared to Gram-negative bacteria. Its antimicrobial activity was attributed to functional destabilisation of the bacterial membrane and the increased sensitivity of bacterial strains to classical antimicrobials. Linalool has been shown to have remarkable activity against periodontopathic and carious bacteria, with minimum inhibitory MIC concentrations ranging from 0.1 to 1.6 mg/mL. Linalool also acts as an anti-lipoperoxidant. Essential oils containing linalool have demonstrated an increase in antioxidant activity, likely due to the synergy between the components. Linalool protects guinea pig brain tissue from oxidative stress caused by hydrogen peroxide at relatively high concentrations (120 mg/kg) and for a long time. Its effects are similar to those of lipoic acid and vitamin E (Aprotosoaie et al., 2014). By increasing the permeability of the skin and mucous membranes, linalool promotes the transdermal penetration of therapeutic agents, making it a useful promoter of absorption in topical preparations. Meanwhile, hydrogel-type preparations appear to absorb more linalool in the *stratum corneum* than emulsions or oil solutions (Aqil et al., 2007). Geraniol has antimicrobial activity against 78 different microorganisms, but *Candida* and *Staphylococcus* are the most frequently tested types of this monoterpene using the broth microdilution technique. Geraniol showed synergistic activity in combination with chloramphenicol, norfloxacin, and tetracycline. MIC values (indicating excellent antimicrobial activity) were ≤600 μg/mL against *E. aerogenes, S. aureus*, *E. coli*, *L. monocytogenes*, *S. epidermidis*, *B. cereus*, S*. typhimurium*, *T. rubrum*, *T. mentagrophytes*, *M. canis*, *M. gypseum*, *C. albicans*, *C. krusei*, *C. glabrata*, *C. tropicalis*, *Curcuma parapsilosis*, *T. asahii*, and *C. neoformans* (Chouhan et al., 2017; Lira et al., 2020). Essential oil from aboveground parts of *A. nobilis* L. subsp. *neilreichii* (Kerner) was tested for composition, antioxidant activity, and antimicrobial activity. Fragranyl acetate (32%) was the main component of the obtained essential oil. The essential oil was found to be effective against the human pathogenic microorganisms tested, showing MIC values in the range of 0.5 to >2 mg/mL and DPPH• (IC_50_ > 0.5 mg/mL) (Bala et al., 2009). High antimicrobial and antioxidant activity classify eugenol as an absorption promoter. Cosmetic formulas for acne skin care recommend eugenol because it improves the complexion of people with acne and psoriasis, reducing the appearance of lesions and speeding up their healing (Kucharska et al., 2024).

In our study, phenolic acids such as gallic acid, 3,4-dihydroxybenzoic acid, 2,5-dihydroxybenzoic acid, vanillic acid, 2,3-dihydroxybenzoic acid, caffeic acid, and chlorogenic acid were identified in the tested *S. officinalis* extract. Other authors have also identified chlorogenic acid and caffeic acid in great burnet, as well as gallic acid, 3,4-dihydroxybenzoic acid, and vanillic acid (Biernasiuk et al., 2015). Not only *S. officinalis* leaves are rich in polyphenolic compounds. For example, in the roots were identified ellagic acid, caffeic acid, p-coumaroylquinic acid, and chlorogenic acid (Tocai et al., 2022), as well as methyl 4-O-β-D-glucopyranosy-5-hydroxy-3-methoxylbenzoate, 3,3′,4′-tri-O-methylellagic acid, fisetinidol-(4α-8)-catechin, and (+)-catechin (Zhang et al., 2012). In flowers were identified, among others, punicalagin gallate, ellagic acid, caffeic acid, and chlorogenic acid (Tocai et al., 2022). In our study, gallic acid was found in the highest concentration. The content of gallic acid in the tested extract may affect its antioxidant and antibacterial potential.

The solvent may have a significant effect on the permeation of natural active substances through the skin (Nowak et al., 2021b; Bertges et al., 2020). In our study, an extract prepared in a 70% *v/v* aqueous ethanol solution was applied to pig skin. This concentration is considered optimal for topical drugs (Nowak et al., 2021a; Tuntiyasawasdikul et al., 2017). Ethanol is a promoter of transepidermal transport, which affects the effectiveness of active substances permeation into the skin. This alcohol can reversibly transform the structure of the laminar system of the lipid matrix of the epidermis, which probably accelerates the diffusion of molecules through the stratum corneum. Ethanol can disrupt the skin’s barrier function by affecting the cells located between the cellular cement. It loosens the lipid layer, increases its fluidity, and consequently the permeation (Jaworska et al., 2011). Tuntiyasawasdikul et al. (2017) demonstrated that applying an ethanol/water mixture increased the permeation of diarylheptanoids from a *Curcuma longa* L. extract compared to a propylene glycol/water solution (Tuntiyasawasdikul et al., 2017). The phenolic acids present in *S. officinalis* may be valuable therapeutic ingredients with antioxidant or antimicrobial properties. This is very important in preparations applied to the skin (Liu et al., 2020). There is also not much information in the literature about the permeation of these compounds through the skin and their accumulation in it, as well as their possible permeation into deeper tissues. In our study, we observed that some phenolic acids contained in the analyzed extracts permeated and accumulated in the skin. For topical use, the ingredients contained in the preparation must reach all layers of the skin (Bertges et al., 2020). Substances that penetrate and accumulate in the skin’s deeper layers may increase its antioxidant potential. It is important because oxidative stress can accelerate skin ageing and disrupt the wound-healing process (Nowak et al., 2021a). Therefore, the topical application of natural plant substances might help improve the endogenous cutaneous protection system (Alonso et al., 2014). However, the plant’s active substances can penetrate tissues to varying degrees, and this parameter depends on many factors, among them the physicochemical properties of the active substances or the use of the vehicle (Nowak et al., 2021b; Klebeko et al., 2021). We presented the permeation results of selected phenolic acids after 24 h. Vanillic acid and 3,4-dihydroxybenzoic acid permeated to a high degree. In another study, 3,4-dihydroxybenzoic acid also permeated through human skin to a high degree from *Epilobium angustifolium* ethanolic extract (Nowak et al., 2021a). Interestingly, we did not identify the caffeic acid in the acceptor fluid after 24-h permeation. Bertges et al. (2020) also did not note the penetration of caffeic acid from coffee extract in the oil-in-water (O/W) emulsion. These authors suggested that this vehicle was not suitable for delivering this bioactive compound to the skin (Bertges et al., 2020). However, an increase in caffeic acid permeation was observed through the pig ear after using liposomes (Katuwavila et al., 2016) and nanostructured lipid carriers (Nitthikan et al., 2018).

It is commonly believed that the permeation of active substances through the skin increases the effectiveness of the applied preparation. On the other hand, the accumulation of active substances in the skin is also very beneficial, especially in the case of topical preparations or cosmetics (Nicolai et al., 2020). We also observed the accumulation of analysed phenolic acids in the skin, with 2,5-dihydroxybenzoic acid, gallic acid, 2,3-dihydroxybenzoic acid, and vanillic acid accumulating in the highest concentration. Bertges et al. (2020) suggested that in the case of cosmetic preparation, lower permeation to the deeper layers will result in a more significant antioxidant effect on the skin (Bertges et al., 2020).

As mentioned, many studies confirm the pharmacological effects of *S. officinalis* extracts, including their antioxidant and antimicrobial potential. However, most of the published research focuses on *Sanguisorbae radix* extracts rather than *Sanguisorbae herba*. The novelty of this study consists of a GC-MS analysis of the composition of the essential oil isolated from the aerial parts of *S. officinalis* and an assessment of the ability of selected phenolic acids contained in the ethanolic extract of great burnet to permeate and accumulate in the skin. However, there are some limitations to this study. The tested extracts were not optimized. No reference antibiotics were used in antibacterial activity tests. Further research focusing, among others, on MIC determination will allow for the optimal selection of extract concentrations. The processes of separating individual oil components are often time-consuming, therefore, it is a great challenge to isolate the oil components to additionally confirm the structures of the main components (identified by mass spectrometry) based on the NMR spectroscopy method.

## 5 Conclusion

The presented study constitutes a preliminary analysis of the usefulness of using ethanolic extracts from *S. officinalis* in preparations applied to the skin. The confirmed antioxidant and antibacterial potential, as well as the ability to permeate and accumulate selected phenolic acids with therapeutic effects in the skin, constitute an introduction to extended research on the use of great burnet extracts in cosmetic and pharmaceutical preparations. In the next stages, it is necessary to optimise the *S. officinalis* extraction process and then extend the analysis of the obtained extract in terms of its health-promoting properties on the skin, including the assessment of its potential toxicity. Additional microbiological tests should also be performed, considering pathogens closely associated with the skin and the MIC assessment. The extended research on an essential oil should include a thorough analysis of its composition and isolation of its components to confirm its structure.

## Data Availability

The original contributions presented in the study are included in the article/Supplementary Material, further inquiries can be directed to the corresponding author.

## References

[B1] Alonso C. Rubio L. Touriño S. Martí M. Barba C. Fernández-Campos F. (2014). Antioxidative effects and percutaneous absorption of five polyphenols. Free Radic. Biol. Med. 75, 149–155. 10.1016/j.freeradbiomed.2014.07.014 25041725

[B2] Antignac E. Nohynek G. J. Re T. Clouzeau J. Toutain H. (2011). Safety of botanical ingredients in personal care products/cosmetics. Food Chem. Toxicol. 49, 324–341. 10.1016/j.fct.2010.11.022 21111022

[B3] Aprotosoaie A. C. Hăncianu M. Costache I. Miron A. (2014). Linalool: a review on a key odorant molecule with valuable biological properties. Flavour Frag. J. 29, 193–219. 10.1002/ffj.3197

[B4] Aqil M. Ahad A. Sultana Y. Ali A. (2007). Status of terpenes as skin penetration enhancers. Drug Discov. Today 12, 1061–1067. 10.1016/j.drudis.2007.09.001 18061886

[B5] Bader A. AlQathama A. Cioni P. L. Ceccarini L. Abdelhady M. I. S. Al-Shareef W. (2022). Essential oil biodiversity of *Achillea ligustica* all. Obtained from mainland and island populations. Plants 11, 1054. 10.3390/plants11081054 35448782 PMC9027389

[B6] Badran M. M. Kuntsche J. Fahr A. (2009). Skin penetration enhancement by a microneedle device (Dermaroller®) *in vitro*: dependency on needle size and applied formulation. Eur. J. Pharm. Sci. 36, 511–523. 10.1016/j.ejps.2008.12.008 19146954

[B7] Bala M. Radhakrishnan T. Kumar A. Mishra G. P. Dobraia J. R. Kirti P. B. (2009). Characterization and biological activity of Achillea teretifolia willd. And *A. nobilis* L. subsp. neilreichii (kerner) formanek essential oils. Turk J. Biol. 33, 129–136. 10.3906/biy-0808-1

[B8] Bertges F. S. Da Penha Henriques Do Amaral M. Rodarte M. P. Vieira Fonseca M. J. Sousa O. V. Pinto Vilela F. M. (2020). Assessment of chemical changes and skin penetration of green Arabica coffee beans biotransformed by *Aspergillus oryza*e. Biocatal. Agric. Biotechnol. 23, 101512. 10.1016/j.bcab.2020.101512

[B9] Bhuiyan M. N. I. Begum J. Sultana M. (2009). Chemical composition of leaf and seed essential oil of *Coriandrum sativum* L. from Bangladesh. Bangladesh J. Pharmacol. 4, 150–153. 10.3329/bjp.v4i2.2800

[B10] Biernasiuk A. Wozniak M. Bogucka-Kocka A. (2015). Determination of free and bounded phenolic acids in the rhizomes and herb of *Sanguisorba officinalis* L. Curr. Issues Pharm. Med. Sci. 28, 254–256. 10.1515/cipms-2015-0083

[B11] Bouazzi S. El Mokni R. Nakbi H. Dhaouadi H. Joshi R. K. Hammami S. (2020). Chemical composition and antioxidant activity of essential oils and hexane extract of *Onopordum arenarium* from Tunisia. J. Chromatogr. Sci. 58, 287–293. 10.1093/chromsci/bmz113 31867630

[B12] Bunse M. Lorenz P. Stintzing F. C. Kammerer D. R. (2020). Characterization of secondary metabolites in flowers of *Sanguisorba officinalis* L. By HPLC-DAD-MS^n^ and GC/MS. Chem. Biodivers. 17, e1900724. 10.1002/cbdv.201900724 32096590

[B13] Byun N. Y. Cho J. H. Yim S. H. (2021). Correlation between antioxidant activity and anti-wrinkle effect of ethanol extracts of *Sanguisorba officinalis* L. Food Sci. Technol. 41, 791–798. 10.1590/fst.10921

[B14] Choi E. S. Kim J. S. Kwon K. H. Kim H. S. Cho N. P. Cho S. D. (2012). Methanol extract of *Sanguisorba officinalis* L. with cytotoxic activity against PC3 human prostate cancer cells. Mol. Med. Rep. 6, 670–674. 10.3892/mmr.2012.949 22710351

[B15] Chouhan S. Sharma K. Guleria S. (2017). Antimicrobial activity of some essential oils—present status and future perspectives. Medicines 4, 58. 10.3390/medicines4030058 28930272 PMC5622393

[B16] Daglia M. (2012). Polyphenols as antimicrobial agents. Curr. Opin. Biotechnol. 23, 174–181. 10.1016/j.copbio.2011.08.007 21925860

[B17] Davies D. J. Ward R. J. Heylings J. R. (2004). Multi-species assessment of electrical resistance as a skin integrity marker for *in vitro* percutaneous absorption studies. Toxicol Vitro 18, 351–358. 10.1016/j.tiv.2003.10.004 15046783

[B18] Değirmenci H. Erkurt H. (2020). Relationship between volatile components, antimicrobial and antioxidant properties of the essential oil, hydrosol and extracts of *Citrus aurantium* L. flowers. J. Infect. Public Health 13, 58–67. 10.1016/j.jiph.2019.06.017 31296479

[B19] Do J. R. Kim K. J. Park S. Y. Lee O. H. Kim B. S. Kang S. N. (2005). Antimicrobial and antioxidant activities of ethanol extracts of medicinal plants. J. Food Sci. Nutr. 10, 81–87. 10.3746/jfn.2005.10.1.081

[B20] Elhawary E. A. Mostafa N. M. Labib R. M. Singab A. N. (2021). Metabolomic profiles of essential oils from selected *rosa* varieties and their antimicrobial activities. Plants 10, 1721. 10.3390/plants10081721 34451766 PMC8398089

[B21] Esmaeili A. Masoudi Sh Masnabadi N. Rustaiyan A. H. (2010). Chemical constituents of the essential oil of *Sanguisorba minor* scop. Leaves, from Iran. J. Med. Plants 9, 67–70.

[B22] Fonseca-Santos B. Corrêa M. A. Chorilli M. (2015). Sustainability, natural and organic cosmetics: consumer, products, efficacy, toxicological and regulatory considerations. Braz J. Pharm. Sci. 51, 17–26. 10.1590/S1984-82502015000100002

[B23] Freije A. Alkhuzai J. Al-Laith A. A. (2013). Fatty acid composition of three medicinal plants from Bahrain: new potential sources of γ-linolenic acid and dihomo-γ-linolenic. Ind. Crops Prod. 43, 218–224. 10.1016/j.indcrop.2012.07.021

[B24] Gawron-Gzella A. Witkowska-Banaszczak E. Bylka W. Dudek-Makuch M. Odwrot A. Skrodzka N. (2016). Chemical composition, antioxidant and antimicrobial activities of *Sanguisorba officinalis* L. Extracts. Pharm. Chem. J. 50, 244–249. 10.1007/s11094-016-1431-0 32214538 PMC7089018

[B25] González-Minero F. Bravo-Díaz L. (2018). The use of plants in skin-care products, cosmetics and fragrances: past and present. Cosmetics 5, 50. 10.3390/cosmetics5030050

[B26] Guimarães A. C. Meireles L. M. Lemos M. F. Guimarães M. C. C. Endringer D. C. Fronza M. (2019). Antibacterial activity of terpenes and terpenoids present in essential oils. Molecules 24, 2471. 10.3390/molecules24132471 31284397 PMC6651100

[B27] Hamedeyazdan S. Fathiazad F. Asnaashari S. (2013). Chemical composition of the essential oil from *marrubium persicum* C. A. Mey. (Lamiaceae). Pharm. Sci. 19, 35–38.

[B28] Haq A. Michniak-Kohn B. (2018). Effects of solvents and penetration enhancers on transdermal delivery of thymoquinone: permeability and skin deposition study. Drug Deliv. 25, 1943–1949. 10.1080/10717544.2018.1523256 30463442 PMC6249612

[B29] Herrera-Pool E. Ramos-Díaz A. L. Lizardi-Jiménez M. A. Pech-Cohuo S. Ayora-Talavera T. Cuevas-Bernardino J. C. (2021). Effect of solvent polarity on the Ultrasound Assisted extraction and antioxidant activity of phenolic compounds from habanera pepper leaves (*Capsicum chinense*) and its identification by UPLC-PDA-ESI-MS/MS. Ultrason. Sonochem 76, 105658. 10.1016/j.ultsonch.2021.105658 34242865 PMC8273200

[B30] Hikmawanti N. P. E. Fatmawati S. Asri A. W. (2021). The effect of ethanol concentrations as the extraction solvent on antioxidant activity of katuk (*Sauropus androgynus* (L.) merr.) leaves extracts. IOP Conf. Ser. Earth Environ. Sci. 755, 012060. 10.1088/1755-1315/755/1/012060

[B31] Islam M. T. Ali E. S. Uddin S. J. Shaw S. Islam M. A. Ahmed M. I. (2018). Phytol: a review of biomedical activities. Food Chem. Toxicol. 121, 82–94. 10.1016/j.fct.2018.08.032 30130593

[B32] Jacobi U. Kaiser M. Toll R. Mangelsdorf S. Audring H. Otberg N. (2007). Porcine ear skin: an *in vitro* model for human skin. Skin. Res. Technol. 13, 19–24. 10.1111/j.1600-0846.2006.00179.x 17250528

[B33] Janus E. Ossowicz P. Klebeko J. Nowak A. Duchnik W. Kucharski Ł. (2020). Enhancement of ibuprofen solubility and skin permeation by conjugation with L-valine alkyl esters. RSC Adv. 10, 7570–7584. 10.1039/d0ra00100g 35492154 PMC9049830

[B34] Jaworska M. Sikora E. Ogonowski J. (2011). Factors influencing the percutaneous penetration of active ingredients. Wiad. Chem. 65, 3–4.

[B35] Katuwavila N. P. Perera ADLC Karunaratne V. Amaratunga G. A. J. Karunaratne D. N. (2016). Improved delivery of caffeic acid through liposomal encapsulation. J. Nanomater 2016, 1–7. 10.1155/2016/9701870

[B36] Khiao In M. Richardson K. C. Loewa A. Hedtrich S. Kaessmeyer S. Plendl J. (2019). Histological and functional comparisons of four anatomical regions of porcine skin with human abdominal skin. Anat. Histol. Embryol. 48, 207–217. 10.1111/ahe.12425 30648762

[B37] Kim H. Y. Eo E. Y. Park H. Kim Y. C. Park S. Shin H. J. (2010). Medicinal herbal extracts of *Sophora radix Acanthopanacis cortex Sanguisorbae radix* and *Torilis fructus* inhibit coronavirus replication *in vitro* . Antivir. Ther. 15, 697–709. 10.3851/IMP1615 20710051

[B38] Kim M. Kim S. R. Park J. Mun S. H. Kwak M. Ko H. J. (2022). Structure and antiviral activity of a pectic polysaccharide from the root of *Sanguisorba officinalis* against enterovirus 71 *in vitro/vivo* . Carbohydr. Polym. 281, 119057. 10.1016/j.carbpol.2021.119057 35074124

[B39] Klebeko J. Ossowicz-Rupniewska P. Nowak A. Janus E. Duchnik W. Adamiak-Giera U. (2021). Permeability of ibuprofen in the form of free acid and salts of L-valine alkyl esters from a hydrogel formulation through strat-m™ membrane and human skin. Materials 14, 6678. 10.3390/ma14216678 34772205 PMC8588543

[B40] Kopečná M. Macháček M. Prchalová E. Štěpánek P. Drašar P. Kotora M. (2017). Galactosyl pentadecene reversibly enhances transdermal and topical drug delivery. Pharm. Res. 34, 2097–2108. 10.1007/s11095-017-2214-3 28664316

[B41] Krauß S. Hammann S. Vetter W. (2016). Phytyl fatty acid esters in the pulp of bell pepper (*Capsicum annuum*). J. Agric. Food Chem. 64, 6306–6311. 10.1021/acs.jafc.6b02645 27458658

[B42] Kucharska E. Zagorska-Dziok M. Bilewicz P. Kowalczyk S. Pełech R. (2024). Use of *Syzygium aromaticum* L. Fermented plant extract to enhance antioxidant potential: fermentation kinetics. App Sci. 14, 4900. 10.3390/app14114900

[B43] Kuntsche J. Bunjes H. Fahr A. Pappinen S. Rönkkö S. Suhonen M. (2008). Interaction of lipid nanoparticles with human epidermis and an organotypic cell culture model. Int. J. Pharm. 354, 180–195. 10.1016/j.ijpharm.2007.08.028 17920216

[B44] Lachowicz S. Oszmiański J. Rapak A. Ochmian I. (2020). Profile and content of phenolic compounds in leaves, flowers, roots, and stalks of *Sanguisorba officinalis* L. Determined with the LC-DAD-ESI-QTOF-MS/MS analysis and their *in vitro* antioxidant, antidiabetic, antiproliferative potency. Pharmaceuticals 13, 191. 10.3390/ph13080191 32806688 PMC7464974

[B45] Liao H. Banbury L. K. Leach D. N. (2008). Antioxidant activity of 45 Chinese herbs and the relationship with their TCM characteristics. Evid-Based Complement. Altern. Med. 5, 429–434. 10.1093/ecam/nem054 PMC2586310 18955214

[B46] Lira M. H. P. Andrade Júnior F. P. Moraes G. F. Q. Macena G. S. Pereira F. O. Lima I. O. (2020). Antimicrobial activity of geraniol: an integrative review. J. Essent. Oil Res. 32, 187–197. 10.1080/10412905.2020.1745697

[B47] Liu J. Du C. Beaman H. T. Monroe M. B. B. (2020). Characterization of phenolic acid antimicrobial and antioxidant structure–property relationships. Pharmaceutics 12, 419. 10.3390/pharmaceutics12050419 32370227 PMC7285200

[B48] Macias Socha C. L. Reyes Cuellar J. C. (2020). Citral nanocontainers applied to guava fruits (*Psidium Guajava* L.) in postharvesting. DYNA 87, 267–276. 10.15446/dyna.v87n212.80496

[B49] Makuch E. Nowak A. Günther A. Pełech R. Kucharski Ł. Duchnik W. (2020). Enhancement of the antioxidant and skin permeation properties of eugenol by the esterification of eugenol to new derivatives. Amb. Express 10, 187. 10.1186/s13568-020-01122-3 33078274 PMC7572966

[B50] Manso T. Lores M. De Miguel T. (2021). Antimicrobial activity of polyphenols and natural polyphenolic extracts on clinical isolates. Antibiot. 30 grudzień 11 (1), 46. 10.3390/antibiotics11010046 PMC8773215 35052923

[B51] Nadeeshani Dilhara Gamage D. G. Dharmadasa R. M. Chandana Abeysinghe D. Saman Wijesekara R. G. Prathapasinghe G. A. Someya T. (2022). Global perspective of plant-based cosmetic industry and possible contribution of Sri Lanka to the development of herbal cosmetics. Evid-Based Complement. Altern. Med. 2022, 1–26. 10.1155/2022/9940548 PMC8916882 35280508

[B52] Nawaz H. Shad M. A. Rehman N. Andaleeb H. Ullah N. (2020). Effect of solvent polarity on extraction yield and antioxidant properties of phytochemicals from bean (*Phaseolus vulgaris*) seeds. Braz J. Pharm. Sci. 56, e17129. 10.1590/s2175-97902019000417129

[B53] Nicolai M. Mota J. Fernandes A. S. Pereira F. Pereira P. Reis C P. (2020). Assessment of the potential skin application of *Plectranthus ecklonii* benth. Pharmaceuticals 13, 120. 10.3390/ph13060120 32532114 PMC7345374

[B54] Niculau E. Ribeiro L. Ansante T. Fernandes J. Forim M. Vieira P. (2018). Isolation of Chavibetol and methyleugenol from essential oil of *Pimenta pseudocaryophyllus* by high performance liquid chromatography. Molecules 23, 2909. 10.3390/molecules23112909 30413007 PMC6278253

[B55] Nitthikan N. Leelapornpisid P. Natakankitkul S. Chaiyana W. Mueller M. Viernstein H. (2018). Improvement of stability and transdermal delivery of bioactive compounds in green robusta coffee beans extract loaded nanostructured lipid carriers. J. Nanotechnol. 2018, 1–12. 10.1155/2018/7865024

[B56] Nour V. Stampar F. Veberic R. Jakopic J. (2013). Anthocyanins profile, total phenolics and antioxidant activity of black currant ethanolic extracts as influenced by genotype and ethanol concentration. Food Chem. 14, 961–966. 10.1016/j.foodchem.2013.03.105 23790874

[B57] Nowak A. Cybulska K. Makuch E. Kucharski Ł. Różewicka-Czabańska M. Prowans P. (2021a). *In vitro* human skin penetration, antioxidant and antimicrobial activity of ethanol-water extract of fireweed (*Epilobium angustifolium* L.). Molecules 26, 329. 10.3390/molecules26020329 33435259 PMC7827182

[B58] Nowak A. Duchnik W. Muzykiewicz-Szymańska A. Kucharski Ł. Zielonka-Brzezicka J. Nowak A. (2023). The changes of antioxidant activity of three varieties of ‘nalewka’, a traditional polish fruit alcoholic beverage during long-term storage. Appl. Sci. 13, 1114. 10.3390/app13021114

[B59] Nowak A. Zagórska-Dziok M. Ossowicz-Rupniewska P. Makuch E. Duchnik W. Kucharski Ł. (2021b). *Epilobium angustifolium* L. Extracts as valuable ingredients in cosmetic and dermatological products. Molecules 26, 3456. 10.3390/molecules26113456 34200200 PMC8201033

[B60] Nzor J. N. Uwakwe A. A. Ogunka-Nnoka C. U. (2024). Comparative analysis of *Anthocleista vogelii* leaf extracts: solvent influence on phytochemical composition, quantitative profile, and in-vitro antioxidant activities. Int. J. Innov. Biochem. Microbiol. Res. 12, 1–7.

[B61] Oke M. A. Bello A. B. Odebisi M. B. Ahmed El-Imam A. M. Kazeem M. O. (2013). Evaluation of antibacterial efficacy of some alcohol-based hand sanitizers sold in Ilorin (North-central Nigeria). Ife J. Sci. 15, 111–117.

[B62] Onocha P. A. Oloyede G. K. Akintola J. A. (2016). Chemical composition, free radical scavenging and antimicrobial activities of essential oil of *Mariscus alternifolius* Vahl. Open Conf. Proc. J. 07 (07), 160–167. 10.2174/2210289201607010160

[B63] Othman L. Sleiman A. Abdel-Massih R. M. (2019). Antimicrobial activity of polyphenols and alkaloids in middle eastern plants. Front. Microbiol. 10, 911. 10.3389/fmicb.2019.00911 31156565 PMC6529554

[B64] Pelc M. Przybyszewska E. Przybył J. L. Capecka E. Bączek K. Węglarz Z. (2011). Chemical variability of great burnet (*Sanguisorba officinalis* L.) growing wild in Poland. Acta Hortic. 925, 97–101. 10.17660/ActaHortic.2011.925.12

[B65] Petri G. Dobson S. Then M. (1991). IR spectroscopic determination of linalyl acetate in the essential oil of *Salvia sclarea* . Planta Med. 57, A138–A139. 10.1055/s-2006-960440

[B66] Radulovic N. Djordjevic N. (2011). Steroids from poison hemlock (*Conium maculatum* L.): a GC-MS analysis. J. Serb Chem. Soc. 76, 1471–1483. 10.2298/JSC110206128R

[B67] Ryu J. Lyu J. I. Kim D. G. Kim J. M. Jo Y. D. Kang S. Y. (2020). Comparative analysis of volatile compounds of gamma-irradiated mutants of rose (*Rosa hybrida*). Plants 9, 1221. 10.3390/plants9091221 32957603 PMC7569881

[B68] Sahoo A. Dash B. Jena S. Ray A. Panda P. C. Nayak S. (2021). Phytochemical composition of flower essential oil of *Plumeria alba* grown in India. J. Essent. Oil-Bear Plants 24, 671–676. 10.1080/0972060X.2021.1965036

[B69] Seo G. E. Kim S. M. Pyo B. S. Yang S. A. (2016). Antioxidant activity and antimicrobial effect for foodborne pathogens from extract and fractions of *Sanguisorba officinalis* L. Korean Soc. Med. Crop Sci. 24, 303–308. 10.7783/KJMCS.2016.24.4.303

[B70] Servi H. Eren Keskin B. Celik S. Budak U. Kababiyik B. (2019). Essential oil and fatty acid composition of endemic *Gypsophila laricina* schreb. From Turkey. Turk J. Pharm. Sci. 16, 220–226. 10.4274/tjps.galenos.2018.49140 32454717 PMC7227973

[B71] Silva B. O. Orlando J. B. Pires C. L. Hiruma-Lima C. A. De Mascarenhas Gaivão I. Perazzo F. F. (2021). Genotoxicity induced by nerol, an essential oil present in citric plants using human peripheral blood mononuclear cells (PBMC) and HepG2/C3A cells as a model. J. Toxicol. Environ. Health A 84, 518–528. 10.1080/15287394.2021.1902443 33761836

[B72] Simon A. Amaro M. I. Healy A. M. Cabral L. M. De Sousa V. P. (2016). Comparative evaluation of rivastigmine permeation from a transdermal system in the Franz cell using synthetic membranes and pig ear skin with *in vivo-in vitro* correlation. Int. J. Pharm. 512, 234–241. 10.1016/j.ijpharm.2016.08.052 27568498

[B73] Son D. Hwang S. Kim M. H. Park U. Kim B. (2015). Anti-diabetic and hepato-renal protective effects of ziyuglycoside II methyl ester in type 2 diabetic mice. Nutrients 7, 5469–5483. 10.3390/nu7075232 26198246 PMC4517009

[B74] Song J. H. Kim S. Y. Hwang G. S. Kim Y. S. Kim H. Y. Kang K. S. (2019). Sanguiin H-11 from *Sanguisorbae radix* protects HT22 murine hippocampal cells against glutamate-induced death. Bioorg Med. Chem. Lett. 29, 252–256. 10.1016/j.bmcl.2018.11.042 30497912

[B75] Sotubo S. E. Lawal O. A. Osunsami A. A. Ogunwande I. A. (2016). Constituents and insecticidal activity of *Deinbollia pinnata* essential oil. Nat. Prod. Commun. 11, 1934578X1601101–90. 10.1177/1934578x1601101228 30508358

[B76] Spigno G. Tramelli L. De Faveri D. M. (2007). Effects of extraction time, temperature and solvent on concentration and antioxidant activity of grape marc phenolics. J. Food Eng. 81, 200–208. 10.1016/j.jfoodeng.2006.10.021

[B77] Srivastava S. Lal R. K. Yadav K. Pant Y. Bawitlung L. Kumar P. (2022). Chemical composition of phenylpropanoid rich chemotypes of *Ocimum basilicum* L. and their antimicrobial activities. Ind. Crops Prod. 183, 114978. 10.1016/j.indcrop.2022.114978

[B78] Su X. D. Ali I. Arooj M. Koh Y. S. Yang S. Y. Kim Y. H. (2018). Chemical constituents from *Sanguisorba officinalis* L. and their inhibitory effects on LPS-stimulated pro-inflammatory cytokine production in bone marrow-derived dendritic cells. Arch. Pharm. Res. 41, 497–505. 10.1007/s12272-018-1035-1 29732490

[B79] Szymański M. Dudek-Makuch M. Witkowska-Banaszczak E. Bylka W. Szymański A. (2020). Comparison of the chemical composition and antioxidant activity of essential oils from the leaves and flowers of *Sambucus nigra* . Pharm. Chem. J. 54, 496–503. 10.1007/s11094-020-02228-5

[B80] Tocai A. C. Memete A. R. Vicaş S. Burescu P. (2021). Antioxidant capacity of *Sanguisorba officinalis* L. And *Sanguisorba minor* scop. Nat. Res. Sustain Dev. 11, 121–133. 10.31924/nrsd.v11i1.072

[B81] Tocai A. C. Ranga F. Teodorescu A. G. Pallag A. Vlad A. M. Bandici L. (2022). Evaluation of polyphenolic composition and antimicrobial properties of *Sanguisorba officinalis* L. And *Sanguisorba minor* scop. Plants 11, 3561. 10.3390/plants11243561 36559673 PMC9785539

[B82] Trivedi P. Klavins L. Hykkerud A. L. Kviesis J. Elferts D. Martinussen I. (2022). Temperature has a major effect on the cuticular wax composition of bilberry (*Vaccinium myrtillus* L.) fruit. Front. Plant Sci. 13, 980427. 10.3389/fpls.2022.980427 36204062 PMC9530925

[B83] Tuntiyasawasdikul S. Limpongsa E. Jaipakdee N. Sripanidkulchai B. (2017). Effects of vehicles and enhancers on the skin permeation of phytoestrogenic diarylheptanoids from *Curcuma comosa* . AAPS Pharm. Sci. Tech. 18, 895–903. 10.1208/s12249-016-0582-3 27380435

[B84] Valgas C. Souza S. M. D. Smânia E. F. A. Smânia Jr. A. (2007). Screening methods to determine antibacterial activity of natural products. Braz J. Microbiol. 38, 369–380. 10.1590/S1517-83822007000200034

[B85] Wang Z. Loo W. T. Wang N. Chow L. W. Wang D. Han F. (2012). Effect of *Sanguisorba officinalis* L on breast cancer growth and angiogenesis. Expert Opin. Ther. Targets 16, 79–89. 10.1517/14728222.2011.642371 22316502

[B86] Wieteska A. Wesołowska A. Jadczak D. (2013). Analysis of the composition of essential oil and extracts obtained from the herb of salad burnet (*Sanguisorba minor* Scop.) by GC/MS method. Univ. Mariae Curie-Skłodowska Lub. – Pol. Eee 23, 1–10.

[B87] Yang J. H. Hwang Y. H. Gu M. J. Cho W. K. Ma J. Y. (2015). Ethanol extracts of *Sanguisorba officinalis* L. suppress TNF-α/IFN-γ-induced pro-inflammatory chemokine production in HaCaT cells. Phytomedicine 22, 1262–1268. 10.1016/j.phymed.2015.09.006 26655409

[B88] Yang J. H. Yoo J. M. Cho W. K. Ma J. Y. (2016). Anti-inflammatory effects of *Sanguisorbae Radix* water extract on the suppression of mast cell degranulation and STAT-1/Jak-2 activation in BMMCs and HaCaT keratinocytes. BMC Complement. Altern. Med. 16, 347. 10.1186/s12906-016-1317-4 27599590 PMC5011966

[B89] Yoshida H. Yamazaki K. Komiya A. Aoki M. Kasamatsu S. Murata T. (2019). Inhibitory effects of *Sanguisorba officinalis* root extract on HYBID (KIAA 1199) – mediated hyaluronan degradation and skin wrinkling. Int. J. Cosmet. Sci. 41, 12–20. 10.1111/ics.12505 30485450

[B90] Yu T. Lee Y. J. Yang H. M. Han S. Kim J. H. Lee Y. (2011). Inhibitory effect of *Sanguisorba officinalis* ethanol extract on NO and PGE_2_ production is mediated by suppression of NF-κB and AP-1 activation signaling cascade. J. Ethnopharmacol. 134, 11–17. 10.1016/j.jep.2010.08.060 20832462

[B91] Zhang L. Koyyalamudi S. R. Jeong S. C. Reddy N. Smith P. T. Ananthan R. (2012). Antioxidant and immunomodulatory activities of polysaccharides from the roots of *Sanguisorba officinalis* . Int. J. Biol. Macromol. 51, 1057–1062. 10.1016/j.ijbiomac.2012.08.019 22944198

[B92] Zhou P. Li J. Chen Q. Wang L. Yang J. Wu A. (2021). A comprehensive review of genus *Sanguisorba*: traditional uses, chemical constituents and medical applications. Front. Pharmacol. 12, 750165. 10.3389/fphar.2021.750165 34616302 PMC8488092

[B93] Zhu A. K. Zhou H. Xia J. Z. Jin H. C. Wang K. Yan J. (2013). Ziyuglycoside II-induced apoptosis in human gastric carcinoma BGC-823 cells by regulating Bax/Bcl-2 expression and activating caspase-3 pathway. Braz J. Med. Biol. Res. 46, 670–675. 10.1590/1414-431X20133050 23969976 PMC3854423

[B94] Zhu H. L. Chen G. Chen S. N. Wang Q. Wan L. Jian S. P. (2019). Characterization of polyphenolic constituents from *Sanguisorba officinalis* L. and its antibacterial activity. Eur. Food Res. Technol. 245, 1487–1498. 10.1016/j.jbiosc.2021.04.003

