# Supplementary material for: Sanguisorba officinalis L. ethanolic extracts and essential oil – chemical composition, antioxidant potential, antibacterial activity, and ex vivo skin permeation study
Source: Front Pharmacol. 2024 Sep 2;15:1390551. doi: 10.3389/fphar.2024.1390551 (PMC11402713; doi:10.3389/fphar.2024.1390551)
Supplement: Supplementary file 1 [file DataSheet1.pdf]

*Supplementary Material*

# *Sanguisorba officinalis* L. ethanolic extracts and essential oil – chemical composition, antioxidant potential, antibacterial activity, and *ex vivo* skin permeation study

**Anna Muzykiewicz-Szymańska*, Anna Nowak, Edyta Kucharska, Krystyna Cybulska, Adam Klimowicz, Łukasz Kucharski**

*** Correspondence:** Anna Muzykiewicz-Szymańska: anna.muzykiewicz@pum.edu.pl

**Table S1.** Results of preliminary analysis of antioxidant activity and total polyphenol content in *Sanguisorba officinalis* L. herb extracts prepared in different concentration of ethanol

| **Ethanol concentration** | **DPPH [mg Trolox/L]** | **ABTS [mg Trolox/L]** | **TPC [mg GA/L]** |
|---|---|---|---|
| 96% *v/v* | 127±2 | 128 ±10 | 128±8 |
| 70% *v/v* | 186±0 | 854±1 | 196±3 |
| 40% *v/v* | 183±0 | 854±2 | 203±5 |

**Table S2.** The result of multivariate analysis of variance of the effect of ethanolic extracts from the *S. officinalis* herb on inhibiting the growth of the tested bacterial strains, $\alpha=0.05$

| Factors | SS | DF | MS | F | p |
|---|---|---|---|---|---|
| **Intercept** | 65802.82 | 1 | 65802.82 | 107433.2 | 0.000000 |
| **S.C.*** | 0.02 | 1 | 0.02 | 0.0 | 0.869186 |
| **E.C.*** | 3399.08 | 3 | 1133.03 | 1849.8 | 0.000000 |
| **Strain** | 264.35 | 9 | 29.37 | 48.0 | 0.000000 |
| **S.C.*E.C.** | 21.28 | 3 | 7.09 | 11.6 | 0.000001 |
| **S.C.*Strain** | 180.32 | 9 | 20.04 | 32.7 | 0.000000 |
| **E.C.*Strain** | 96.08 | 27 | 3.56 | 5.8 | 0.000000 |
| **S.C.*E.C.*Strain** | 52.05 | 27 | 1.93 | 3.1 | 0.000004 |
| **Error** | 98.00 | 160 | 0.61 | | |

*S.C. - solvent concentration, *E.C. – extract concentration
SS – Sum of Squares; DF – Degrees of Freedom; MS – Mean Square; p – p-level

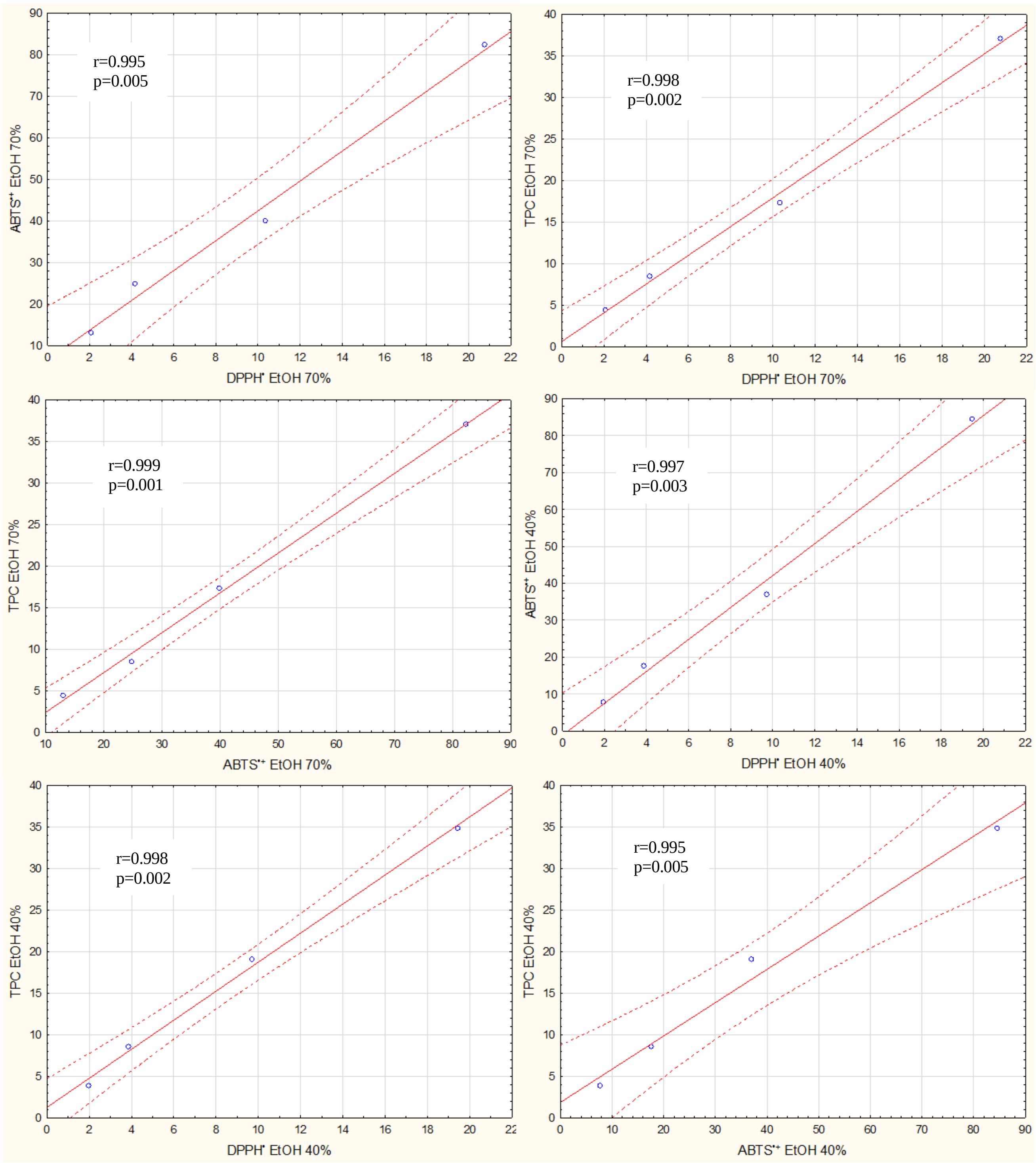


**Figure S1.** Correlations (r) between antioxidant activity determined by the DPPH$^{•}$ and ABTS$^{•+}$methods as well as the total polyphenol content (TPC) in *S. officinalis* extracts prepared in 70% and 40% ethanol, α=0.05

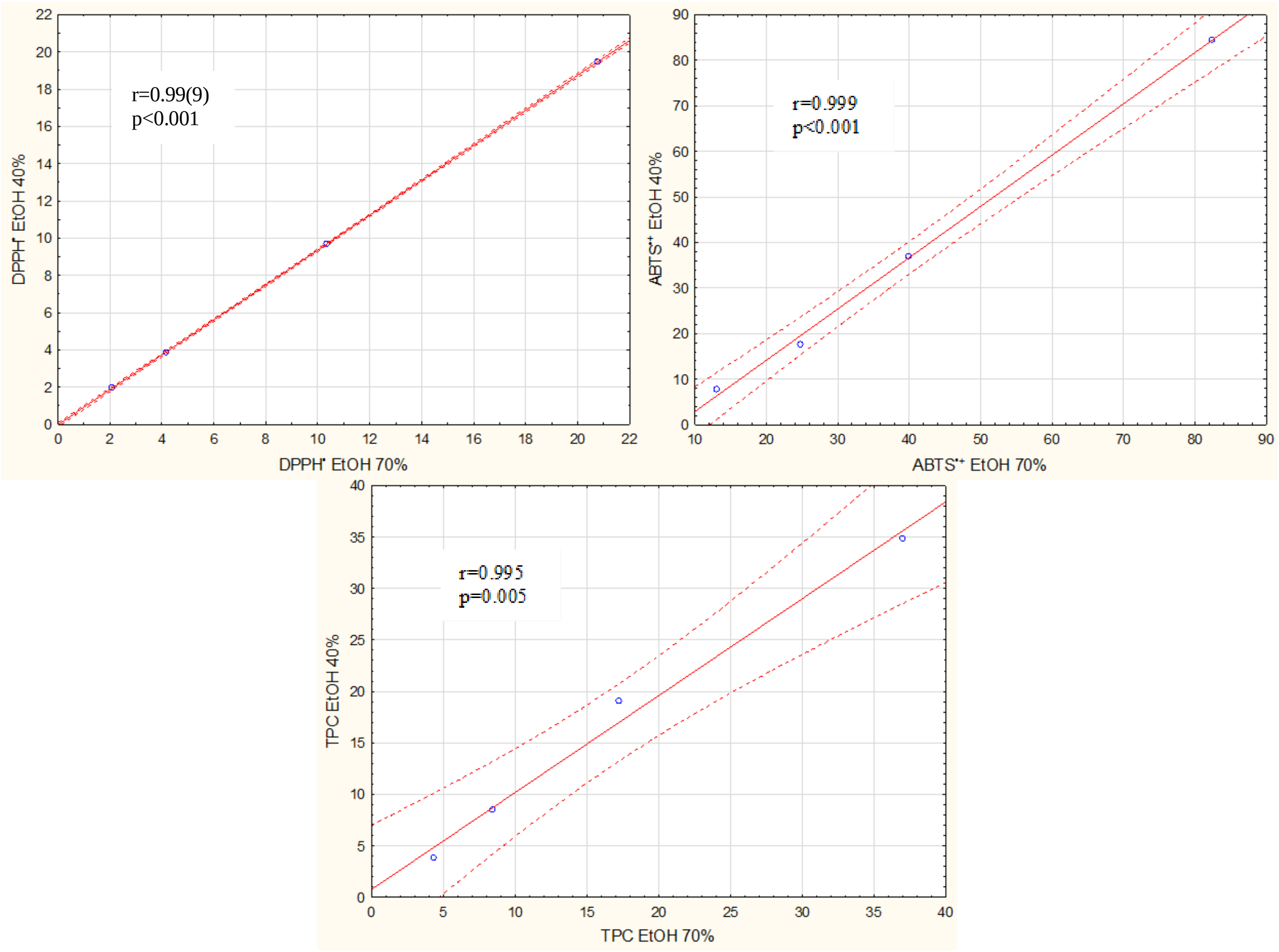


**Figure S2.** Correlations (r) between the activity of *S. officinalis* extracts prepared in different ethanol concentrations (70% or 40% *v/v*), assessed by DPPH$^{•}$ and ABTS$^{•+}$ and Folin-Ciocalteu (TPC) methods, α=0.05

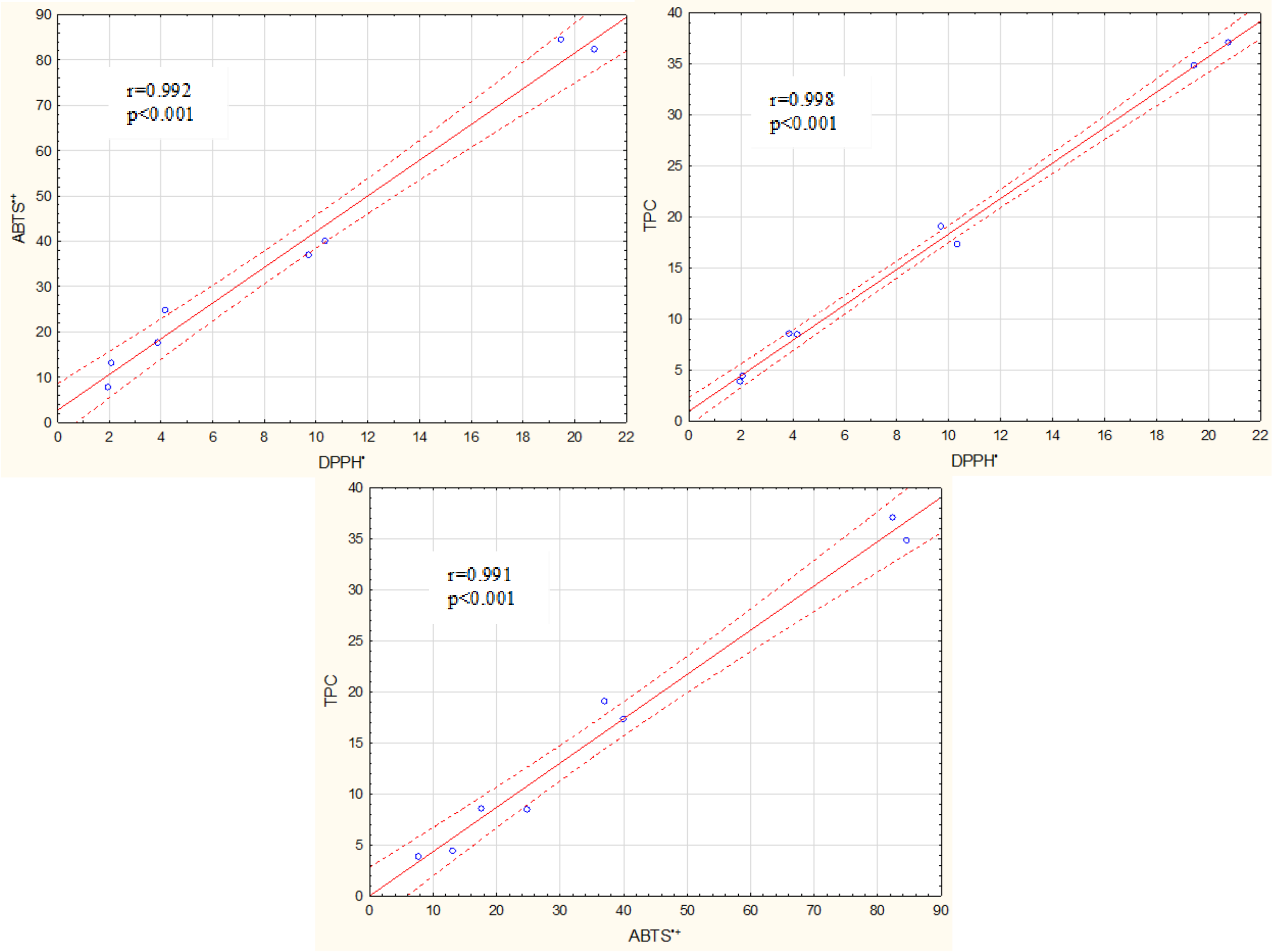


**Figure S3.** Correlations (r) between the results obtained by individual methods (DPPH$^{\bullet}$, ABTS$^{\bullet+}$, and Folin-Ciocalteu (TPC)), regardless of the ethanol concentration used to extraction, α=0.05
